# Modelling, simulation and performance evaluation of the IEEE 802.11e protocol with station mobility

**DOI:** 10.7717/peerj-cs.1457

**Published:** 2023-07-13

**Authors:** Estefanía Coronado, Valentín Valero, M. Emilia Cambronero, Luis Orozco-Barbosa

**Affiliations:** 1 Departamento de Sistemas Informáticos, Universidad de Castilla La Mancha, Albacete, Spain; 2 i2CAT Foundation, Barcelona, Spain

**Keywords:** IEEE 802.11, QoS, Colored Petri Nets, Performance evaluation

## Abstract

In this article, we present a parameterized Colored Petri Net (CPN) model of the IEEE 802.11e protocol for wireless communications with mobile stations. CPNs provide a graphical model for the modeling and analysis of concurrent systems, which can be parameterized by the use of constants, and thus they allow us to create more flexible models. Our CPN model captures the protocol’s behavior, and the specific parameters used for the 802.11e protocol and the scenarios to be evaluated are captured by the CPN parameters. The model presented is flexible enough to cover full customization of traffic types, user mobility and collision avoidance protocols. In this model, there is an access point (AP) which is visible to all the stations, and we assume that due to physical restrictions, there are two range groups. All the stations in the same range group are visible to each other. The impact of mobility is then analyzed by studying a situation in which the stations move in a controlled way to the same range group. The simulation results demonstrate the impact on network performance for sensitive and insensitive traffic types, as well as the role of the RTS/CTS protocol in collision avoidance, especially when users are located in different regions. Specifically, we show how the performance improves in the different scenarios when the stations move to the same area, where they can see each other, and we also study the impact on the performance for each type of traffic.

## Introduction

In the last few years, society has progressively adopted a lifestyle which relies upon constant communication. Telework, autonomous driving, social networks and video applications are just a few examples motivating this transformation, which has been accompanied by a sustained evolution of communication networks. Wireless networks are the target of these application scenarios, including Wireless Local Area Networks (WLANs), vehicular networks, cellular networks, wireless sensor networks, *etc.* Unlike the rest, which are usually tailored to specific applications (*e.g.*, Internet of Things, manufacturing, *etc.*), WLANs are widely used for multiple device types without complex deployment options, and not only for day-to-day Internet connectivity, but also for more advanced applications such as security and surveillance, autonomous vehicle communications, and real-time video delivery services, where users are in constant movement, generating more packet collisions and greater performance drops.

In this regard, it is crucial to carry out deep analyzes of wireless networks with different network topologies, radio channels, and mobility patterns, and use a wide set of protocols and key parameters in the performance evaluation process. Modeling and simulation have made it possible to focus on specific aspects of network behavior, allowing us to evaluate a wider range of scenarios and consider numerous users without incurring the great cost associated with deployment and operation, on experimental testbeds. This is especially true for mobility scenarios, given the difficulty of isolating the radio interference, ensuring reproducible results, and evaluating mobility patterns with real users in these environments.

The above use cases require the network to support different traffic requirements. For instance, autonomous vehicle communications could impose low delay requirements for camera stream analysis, but less stringent needs may be related to Internet surfing or touristic information acquisition in the car. Although the performance of WLANs governed by the IEEE 802.11 standard has been extensively studied with strong mobility factors (Muktiarto, Perdana & Negara, 2018; Pal, Tripathi & Kumar, 2019), especially with the emergence of management strategies such as software-defined networking (SDN) (Gilani et al., 2017; Gonzalez Orrego & Urrea Duque, 2017), to the best of our knowledge little research effort has been devoted to evaluating such factors in high node density scenarios, such as the vehicular use cases, with the management of a variety of traffic types (*i.e.,* IEEE 802.11e). In this regard, both in city-wide and highway environments, users may transition across several network coverage areas along their path, experiencing varying signal strength, and impacting the performance of other applications types consumed by other non-detected users (Xiang et al., 2020; Mouton et al., 2015). Similarly, other works analyze the Quality of Service (QoS) mechanisms allowing the IEEE 802.11 networks to manage various applications requirements with random topology configurations (Farej & Jasim, 2020). Despite the insights provided into throughput and retransmission attempts, the aspect of user mobility, coverage areas and hidden nodes is not addressed in the study.

This article extends the analysis of the QoS mechanisms performed in one of our previous works (Coronado et al., 2021). In that work, we presented a parameterized Colored Petri Net (CPN) model for the IEEE 802.11e protocol, in which no mobility was considered, and all the stations were hidden from each other (*i.e.,* the stations could not sense when another one was starting a transmission). Thus, we analyzed several scenarios in which there were massive collision chains and high transmission delays. Our main goal was to evaluate the protocol’s effectiveness and the collision avoidance mechanisms defined by the IEEE 802.11 standards. By contrast, the present work aims to analyze the impact of mobility in controlled scenarios. That is to say, this work extends our previous results by focusing on mobility, and the impact of users converging in the same area, where they can see each other. Throughout this study, we consider a specific model of mobility in order to obtain reproducible methods. This approach should allow us to evaluate the impact of the various protocol parameters and mechanisms and the geographical distribution of the stations on the network operation performance.

**Figure 1:**
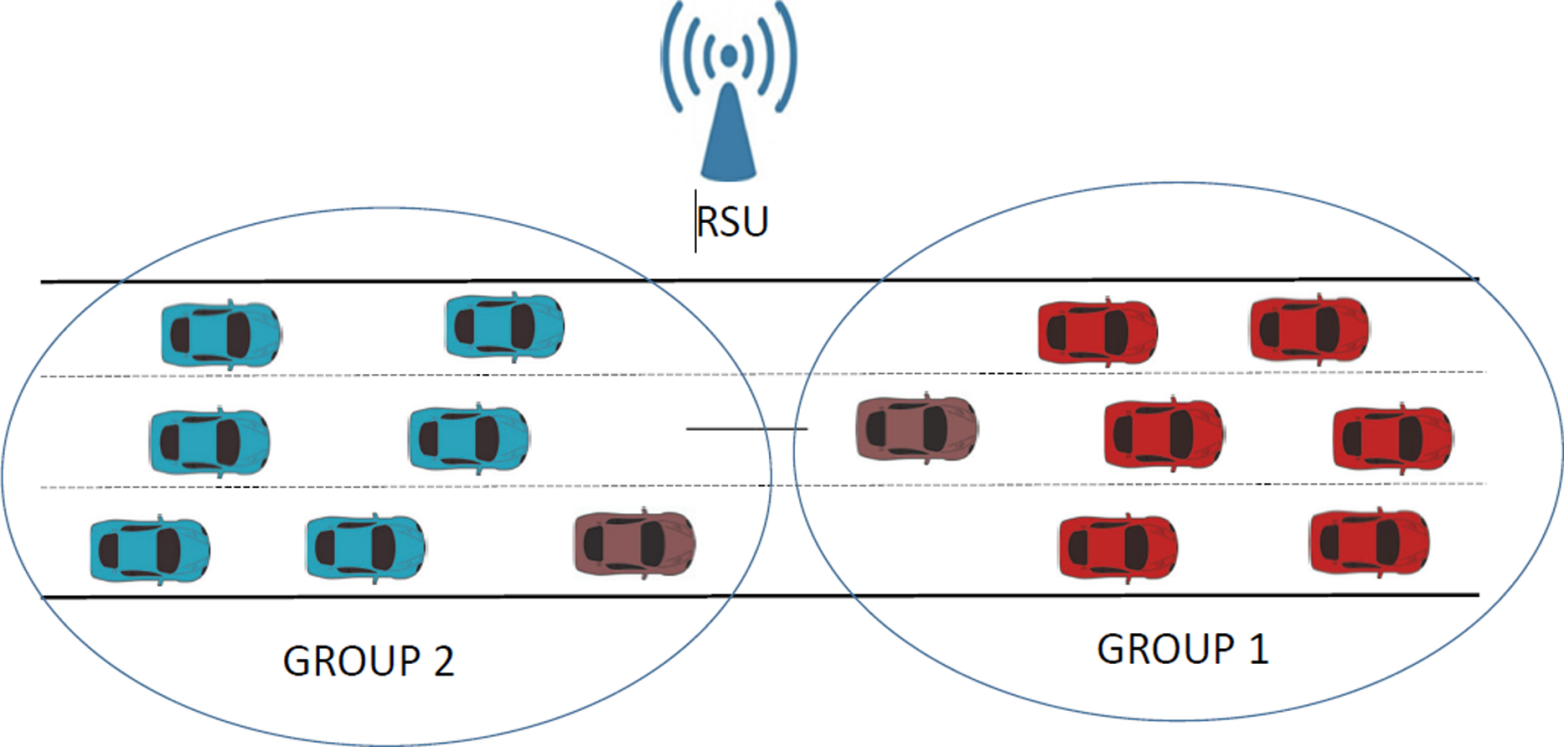
Example of mobility in a vehicular network.

The main contributions of our work are the development of a parameterized CPN model and the study of various scenarios that allow us to evaluate the effectiveness of different protocol mechanisms with regard to the performance of IEEE 802.11-compliant wireless networks. The proposed model should prove an invaluable tool for the network analyst, as the model can be easily configured to set various protocol parameters and enable/disable multiple protocol mechanisms. The protocol parameters that can be configured for different traffic classes include packet size and transmission rates. As for the protocol mechanisms, our CPN model includes the setting of various traffic priorities and enabling/disabling collision avoidance protocols. It is worthwhile pointing out that the CPN model can support any number of visibility groups (*i.e.,* coverage areas), and only the CPN page related to mobility needs to be modified in order to consider any number of groups and the mobility scenario of interest. Note that our model’s ability to parameterize all of these protocol parameters grants it significant flexibility, enabling the evaluation of numerous scenarios. Although in this article, for reasons of space, we have only presented some of these scenarios, other researchers investigating potential issues in these networks could greatly benefit from the model. In particular, in this article, our numerical study considers two visibility groups, where all the stations in the same group can see the channel as busy when some transmission in that group is in progress. However, the stations in one group cannot see the communications taking place from stations in the other group (*i.e.,* ongoing transmissions from stations in other coverage areas). Furthermore, we consider a situation where all the stations progressively move to the same location. Figure 1 depicts a scenario in vehicular communications, in which vehicles from group 2 move progressively towards group 1, with both connected to the same Road side unit (RSU), which plays the role of Access Point (AP) in this specific scenario.

The article is structured as follows. The most relevant related works are described in the “Related Work” section. The section entitled “IEEE 802.11-Based Wireless Networks” presents an overview of the IEEE 802.11e protocol and the motivations for analyzing the impact of mobility. CPNs are described in the section named “Colored Petri Nets”, while the “CPN Model” section presents the CPN model proposed in this article. The performance evaluation and the interpretation of the results in specific scenarios are presented in the “Evaluation” section. Finally, the conclusions and future lines of work are presented in the “Conclusions and Future Work” section.

## Related Work

There are many works devoted to analyzing the IEEE 802.11 protocols. In 2001, the IEEE 802.11e standard, which is the main focus of our article, was published as an amendment to the IEEE 802.11 standard to define QoS for wireless LAN applications by modifying the MAC layer. In this section, we focus on those works that have analyzed these standards using formal methods.

Hassan, Vu & Sakurai (2011) present an analytical model to evaluate the performance of the IEEE 802.11 distributed coordination function (DCF) MAC protocol. With the help of this analytical model, the researchers compute the mean and standard deviation of packet delays and the packet delivery ratio (PDR) at the MAC layer in an unsaturated network formed by moving vehicles on a highway. The results indicate that packet collisions caused by transmissions from hidden terminals are the primary cause of the low PDR of the DCF protocol. The authors propose a novel protocol based on DCF that uses an out-of-band busy tone as a negative acknowledgement to provide an efficient solution to this problem. They extend the analytical model to the enhanced protocol and show that it preserves predictive accuracy. On the basis of the evaluation of the proposed enhanced protocol, the authors concluded that it increases the PDR by up to 10% and increases the supported vehicle density by up to two times for a range of packet arrival rates while maintaining a delay below the threshold. However, the authors of this study do not address how the mobility of the stations affects the study.

Alasmary & Zhuang (2012) evaluate the impact of mobility on IEEE 802.11p infrastructure-less vehicular networks. The authors claim that broadcast and unicast scenarios exhibit an unfairness problem due to relative speed, so they propose two dynamic contention window mechanisms to alleviate network performance degradation caused by high mobility. While the first scheme adapts the priority according to the number of neighboring nodes, the second scheme determines priority based on node relative speed. Several performance metrics are measured, including packet delivery ratio, throughput, and delay. In this study, extensive simulation results provide evidence of the significance of mobility for IEEE 802.11p MAC performance, the unfairness problem in vehicle-to-vehicle communications (Touil, Ghadi & El Makkaoui, 2021) (V2V), and the effectiveness of the proposed MAC schemes. For this purpose, the authors model scenarios using dynamic priority management schemes and simulate them using Network Simulator (NS2) (Varadhan, 2003). In contrast, we develop a richer, more flexible, and more scalable parameterized model based on CPNs, where RTS/CTS handshakes are enabled or disabled, and four types of prioritized traffic are considered.

Molina-Masegosa, Gozalvez & Sepulcre (2020) perform an in-depth analysis of two technologies for V2X (Vehicle to Everything) communication, one based on IEEE 802.11p (such as DSRC Xu et al., 2004 or ITS-G5 Karoui, Freitas & Chalhoub, 2020) and the other on LTE-V2X, also known as Cellular V2X or C-V2X (Fallgren et al., 2021). This research studies how the generation and size of V2X messages can significantly impact the operation and performance of the MAC layer under varying message traffic scenarios. This variability of the message size can negatively affect performance. Their focus is on periodic and aperiodic messages of constant or variable size, based on standardized ETSI Cooperative Awareness Messages (CAMs). The evaluation results show that IEEE 802.11p provides better performance than LTE-V2X unless the channel load is extremely low when transmitting periodic messages varying in size. Our article also analyzes different message sizes, but we also take into account other parameters, such as the number of stations, datarate, and whether RTS/CTS is used.

Aziz (2021) also propose a formal approach, but they focus on analyzing the effects of single failures on the Open Charge Point Protocol for electric vehicle charging. For this purpose, they define process algebraic specifications for analyzing how single failures can impact protocol safety and security. Specifically, they use a variation of pi-calculus to specify a general mutation function for introducing changes in the protocol messages, communication channel names, and input action duration. A case study of an electric vehicle charging protocol illustrates the proposed framework. In the analysis, different kinds of mutants are observed, some of which were indistinguishable from the unmutated cases, others did not influence the system, and others had implications for the safety and security of the protocol.

Entezari-Maleki et al. (2021) propose a model based on stochastic reward nets (Muppala, Ciardo & Trivedi, 1994) (SRNs) to evaluate the performance metrics on multihop wireless networks, including sending and receiving rates of a node as well as the collision probability. SRN is used to model a single node as a general template applied to any wireless node. The main goal of this work is to analyze all the transmission effects of all neighboring nodes, while ignoring those whose transmission does not affect the node under study. They claim that the proposed SRN model allows specifying any arbitrary topology and evaluates the mean queue size, collision probability, and sending and receiving rates among the nodes. Different aspects of MAC and the physical layer of wireless networks, such as hidden and exposed node problems, collisions, transmissions, interference, and carrier sense ranges, are discussed. Validating the results obtained in two scenarios with the results from a discrete-event simulator demonstrates the applicability and accuracy of the proposed SRN model. However, they do not consider how the mobility of wireless nodes affects performance, but they plan to analyze this issue in future work. The research carried out by Farej & Jasim (2020) goes one step further, analyzing *via* simulation (*i.e.,* using the OPNET modeler) the impact of random topologies when considering different characteristics of the IEEE 802.11 standard for QoS support. In this sense, throughput, delay and the level of retransmissions are evaluated with different physical configurations, including the number of streams, with up to 18 nodes. However, this work does not consider user mobility, the consequences of isolated coverage areas or the issue of hidden nodes.

Ding et al. (2022) introduce the variable Petri net (VPN), a new Petri net model for modeling and evaluating mobile systems, including wireless communications systems. Among the sample models provided in their work, they include a study case similar to the one reported in this work. According to the authors, models developed using CPNs do not offer the flexibility necessary to naturally model the components that leave and join a system. As can be seen here, from our research efforts, we have introduced a parameterized CPN model for the modeling of mobile wireless networks. Furthermore, our model includes the definition of a set of relevant parameters for many features defining the communication protocol.

Recently, Raviglione et al. (2021) presented an experimental evaluation of IEEE 802.11-based V2I communications. They show the results of an extensive field test campaign of non-mmWave (IEEE 802.11p and 802.11ac) and mmWave IEEE 802.11 (IEEE 802.11ad) technologies for V2I communications. Specifically, they evaluate the IEEE 802.11p, IEEE 802.11ac, and IEEE 802.11ad protocols, showing their advantages and disadvantages. They assess their performance by measuring the connection stability, the received signal level, and the Round Trip Time and UDP throughput in both Line-Of-Sight and Non-Line-Of-Sight conditions. The results indicate that IEEE 802.11ac and IEEE 802.11ad are the most promising technologies, although not specifically designed for vehicular communications. IEEE 802.11ad, with its use of the 60 GHz spectrum, can offer high throughput and low latency in dense urban scenarios for a few tens of meters between communicating nodes. The authors do not consider the IEEE 802.11e protocol in this study.

In another recent related paper by Sosa et al. (2022), the authors introduce a Petri net-based specification and execution model to manage ad-hoc distributed systems. They illustrate their applicability to various use cases including the communications services of vehicular ad hoc networks (VANETs). In particular, they model the spectrum sensing tasks performed by the vehicles and transmitted to the roadside units (RSUs): an essential task in VANET systems to ensure not only the connectivity of the vehicle, but more importantly the resource management of the network links, *i.e.,* available bandwidth. However, their study does not model the potential conflicts that may arise when a large number of vehicles attempt to access the wireless channel.

From the above literature review, it is clear that none of the works use Colored Petri Nets as a formal method for specifying and analyzing the IEEE 802.11 protocol in the context of mobile systems. Our main contributions in this work are:

• The design of a parameterized colored Petri net (CPN) model for the IEEE802.11e standard with station mobility.
• The implementation and validation of the CPN model using CPN Tools (CPN Tools, 2021).
• An in-depth analysis of various protocol mechanisms introduced by the standard, mainly the RTS/CTS mechanism and the IEEE802.11e priority in mobility scenarios.
• A numerical evaluation in terms of multiple metrics of interest, namely throughput, percentage of lost messages, RTS collisions (when RTS/CTS is used), and transmission times.
• Analysis of the effectiveness and recommended threshold setting of the RTS/CTS mechanism under different loads in single and multi-priority traffic scenarios.

## IEEE 802.11-based Wireless Networks

The research into, and development of, wireless communication technologies have allowed the implementation of mobile wireless communication networks. From Internet of Things (IoT) devices to Intelligent Transportation Systems (ITSs), IEEE 802.11-compliant wireless networks play a fundamental role in the successful deployment of mobile services. In this work, we focus on analyzing the connectivity of wireless devices on the move within a wireless infrastructure. More specifically, we develop and evaluate the performance of the IEEE 802.11 Media Access Control (MAC) protocol in various scenarios. Since nowadays these networks have to provide different QoS guarantees, our study includes an analysis of the Enhanced Distributed Channel Access Function (EDCA) enabled by the IEEE 802.11e amendment.

### IEEE 802.11e capabilities

The MAC protocol used in the 802.11 (Pazzi, Zhang & Boukerche, 2010) standards is the so-called DCF function, which makes all stations in the network compete to access the channel with the same priority. Under this protocol, before starting a transmission, the stations must sense the medium as free for a period given by the DCF Interframe Space (DIFS). If after this period the medium is still busy, they must initiate a Backoff procedure, which is performed by a channel access arbitration algorithm when two or more stations attempt to access the channel simultaneously, generating a collision. When this happens, these stations receive a signal to start the Backoff procedure and stop the transmission. The Backoff procedure delays the transmission for a certain time, thus allowing other stations to complete their transmissions.

**Figure 2:**
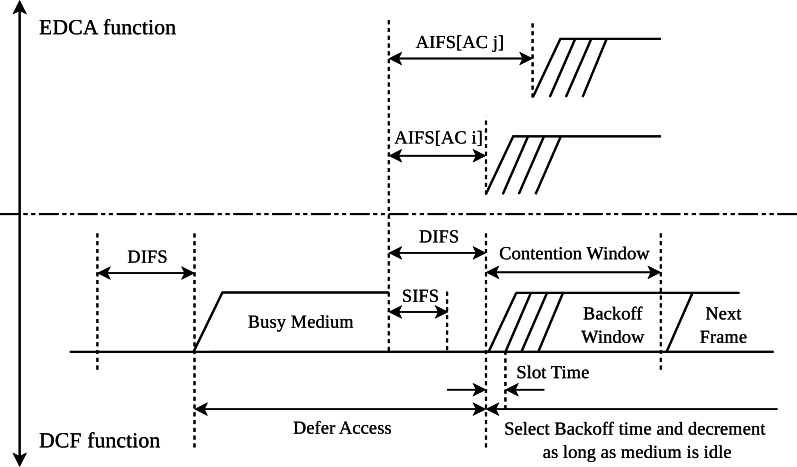
Interframe spaces in DCF and EDCA.

Every time the Backoff procedure is triggered, the waiting times increase exponentially to reduce the collision probability with other stations. To calculate the waiting time, the algorithm selects a random value in the interval [0, *CW* − 1]. The value selected in this interval is directly determined by the contention window (CW). The CW is a parameter defined by the specific physical layer used and is given by the low and upper bounds *CW*_*min*_ and *CW*_*max*_, respectively. The algorithm first sets the size of *CW* to *CW*_*min*_, and then increases it after *n* erroneous transmissions following a sequence of 2^*n*^ until the values reaches the upper bound *CW*_*max*_. In this case (*i.e.,* if the value of *CW* reaches the upper bound), the station must discard the frame. Otherwise, every time that the station senses the channel as idle for a *DIFS* period, one unit is subtracted from the current value. Therefore, this mechanisms allows the station to start the transmission when the counter decreases to zero. Nevertheless, this mechanism gives the same priority to all users and applications. This process is shown in the bottom part of Fig. 2, where the Short Interframe Space *(SIFS)* defines the period of time that can be employed by packets with high priority, such as *ACKs*. Notice that *ACK* control packets are specified by the standard in unicast transmissions to confirm the successful reception of data packets. Consequently, the waiting time of such control frames is lower than the one of data frames to avoid having a long time between the transmission of the data frame and its confirmation, and making the sender (erroneously) retransmit the frame if the *ACK* is not received after a given period of time.

The original IEEE 802.11 standard and the base DCF method do not provide any QoS assurance. To address this issue, the IEEE 802.11e amendment (LAN/MAN Standards Committee of the IEEE Computer Society, 2005) presented the Enhanced Distributed Channel Access(EDCA) function as an evolution of DCF to bring differentiated channel access and prioritize traffic categories, thus introducing the QoS level required. In this regard, EDCA introduces four traffic access categories (ACs), namely Voice (VO), Video (VI), Best Effort (BE) and Background (BK). While voice traffic has the highest priority and the smallest waiting time, background traffic is defined with the lowest priority. Each AC makes use of its own traffic queue and has its own set of medium access parameters: Arbitration Interframe Space (AIFS), Transmit Opportunity (TXOP), and contention window (CW). It should be noted that the stations employing DCF (*i.e.,* without QoS support) maintain the use of the aforementioned DIFS period, while the QoS stations use the AIFS one. Conversely, the TXOP enables the transmission of several consecutive frames of the same AC without initiating a new sensing channel period. To ensure compatibility with the stations using DCF while respecting the traffic priorities, the standard establishes a fixed set of values for EDCA (Table 1). Variable *σ* in Table 1 represents the duration of the slot time given by the physical layer. As can be seen in the table, EDCA specifies different waiting times for each type of traffic.

**Table 1:** Medium access parameters in IEEE 802.11e.

**AC**	**CW_min_**	**CW_max_**	**AIFS**	**TXOP**
**BK**	*acW*_*min*_	*aCW*_*max*_	*SIFS* + 7⋅*σ*	–
**BE**	*aCW*_*min*_	*aCW*_*max*_	*SIFS* + 3⋅*σ*	–
**VI**	$ \frac{(aC{W}_{min}+1)}{2} -1$	*aCW*_*min*_	*SIFS* + 2⋅*σ*	3.008
**VO**	$ \frac{({aCW}_{min}+1)}{4} -1$	$ \frac{({aCW}_{min}+1)}{2} -1$	*SIFS* + 2⋅*σ*	1.504

The timing diagram for the different traffic classes in EDCA is shown in Fig. 2. Note that a station must sense the channel idle for a period equal to AIFS before attempting a transmission. Similarly to DCF, a station must follow the Backoff algorithm if it finds the medium busy (Bianchi, 2000).

### RTS/CTS as carrier-sensing protocol

**Figure 3:**
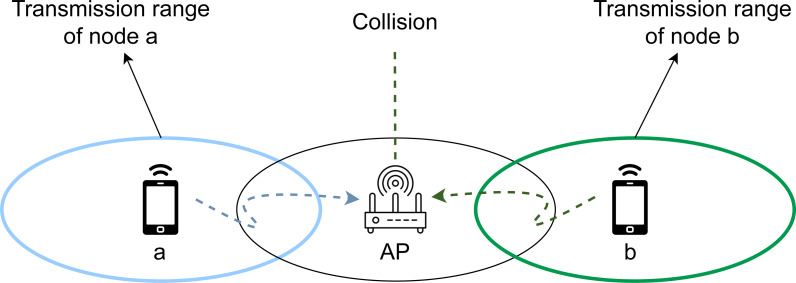
The hidden node problem in wireless networks.

The IEEE 802.11 standard introduces Request to Send/Clear to Send (RTS/CTS) as a carrier sensing protocol for collision avoidance to solve the problems introduced by the presence of hidden nodes. Hidden nodes can be described as a set of two or more stations that are not within the coverage range of each other. Therefore, the standard channel sensing protocols are not able to detect whether these hidden stations are about to start a transmission, which consequently generates a huge number of collisions and retransmissions, especially in scenarios containing stations transmitting various traffic types, characterized by different requirements and channel access waiting times. An example of this problem can be observed in Fig. 3, where the coverage ranges of stations a and b do not have common areas despite being connected to the same access point (AP), therefore causing a collision when both of them start a new transmission.

**Figure 4:**
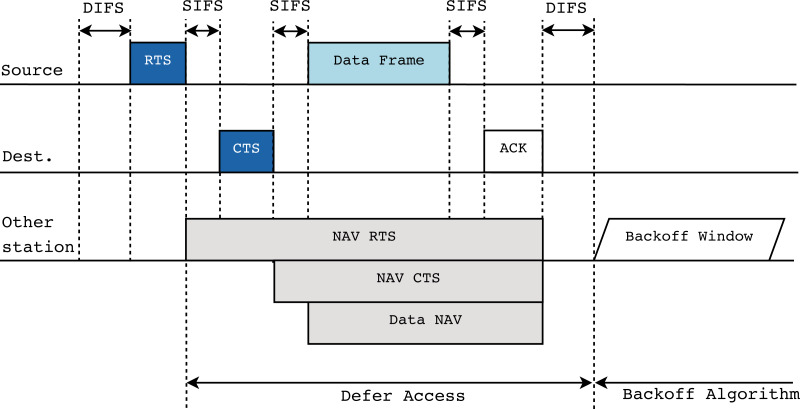
RTS/CTS protocol description in IEEE 802.11.

The RTS/CTS protocol aims to serve as a solution to this problem. The main objective is to allow any station to announce its intention to start a new transmission, even if some stations in the network are not in the coverage area of the first one. The message exchange of the protocol is displayed in Fig. 4. By focusing on the previous example (Fig. 3), and in particular, on station a (in blue) starting a transmission, the protocol is established prior to this event as follows. Station b, which is the source of the new transmission, must first send an RTS frame to the AP. When the AP receives this control packet from any station, it must broadcast a CTS frame to all the remaining stations in the network to inform them about the imminent beginning of the transmission, therefore preventing them from starting a new one during a given period of time. This period is provided by the Network Allocation Vector (NAV), which is included in the RTS/CTS frames, and indicates an estimation of the time required by the source stations to occupy the channel. Then, the stations must set their own NAV time to the received one, thus avoiding channel sensing during this period.

As described above, it can be seen how the RTS/CTS protocol is of great help in scenarios with hidden nodes and how it greatly contributes to performance improvement and collision reduction. This is especially important when various types of traffic are present in the network, since in the event that more sensitive traffic is lost in collisions, the performance and the user experience suffer greatly.

## Colored Petri Nets

A Petri net (Peterson, 1981) is a directed graph with two types of node: places and transitions. Places are drawn as circles, and they usually represent states or system conditions, while transitions are depicted as rectangles and represent actions that change the system state. Places can have a marking, which is a natural number that represents the place state (number of tokens in the place), for instance, the number of messages waiting to be sent in a queue.

Arcs can only be of two types, PT-arcs (place to transition arcs) or TP-arcs (transition to place arcs), and they can have a weight associated to them. A transition can be fired (executed) when all its precondition places have at least a number of tokens equal to the weight of the arcs connecting these places with the transition. In that case, when the transition is fired, for each precondition place, a number of tokens equal to the weight of the arc connecting the place with the transition are removed from the place, and for each postcondition place, a number of tokens equal to the weight of the arc connecting the transition with the place are produced in the place.

Colored Petri nets (CPNs) (Jensen & Kristensen, 2009) are a timed extension of Petri Nets, in which the tokens in the places are typed, *i.e.,* they belong to a certain color set, which is a data type in CPN terminology. For instance, we can have places with the color set *INT*, whose tokens are integers, or a Cartesian product of two or more color sets, as *INT*2 = *INT* × *INT*. We can also have places with no attached information (as in plain Petri Nets), in this case the associated color set is *UNIT*.

Places can also be timed or untimed. Timed places have timed tokens, which means that every token in these places must have an associated timestamp. We use a discrete time model, so timestamps are natural numbers, and there is a global clock that represents the total time elapsed in the system. The token timestamp indicates the time at which the token can be used to fire a transition. When no transition can be fired at a certain time, time elapsing occurs, until reaching a global time in which some transition is fireable.

In this article, we use CPN Tools (CPN Tools, 2021), which is a widely-used tool for creating, editing, analyzing and simulating CPNs. The notation used throughout the article is the one used in CPN Tools. For instance, the number of tokens in a place is drawn in a green circle beside the place, and the specific values are indicated using the notation *n*‘*v* (*n* instances of *v*). The union of colors (values) is denoted by the symbol ‘++’ for untimed places and ‘+++’ for timed places. Thus, the marking 5‘6@7 +  +  + 3‘1@10 contains five integer tokens with value 6 and timestamp 7, and three integer tokens with value 1 and timestamp 10.

Arc inscriptions are now expressions, which are constructed using variables, constants, operators and functions. Arc expressions must evaluate to a multiset of colors in the color set of the attached place. In order to fire a transition there must be a binding^1^ 1A binding for a transition is an assignment of values to the variables used in the PT-arcs of the transition.of the variables used in the expressions of the PT-arcs such that in all its precondition places we have at least the tokens indicated by the evaluation of the corresponding arc inscription expression. These tokens will be removed from the precondition places when the transition is fired, and new tokens are produced in the postcondition places, according to the corresponding arc expressions.

Furthermore, the expressions in both PT-arcs and TP-arcs can be timed, with the syntax *expression@t*. In the case of PT-arcs, the tokens used to fire the transition can be used in advance, *t* time units before their associated timestamp. On the other hand, in the case of TP-arcs, the new tokens produced are aged by the time *t*, with respect to the current global time. Transitions can also have guards and priorities. A transition guard is a Boolean expression, and it must be evaluated to true for the transition to be *fireable*. Priorities are integer values used to resolve choices, when some transitions are simultaneously fireable. We only use two levels of priority, namely *P_NORMAL* and *P_HIGH*, the latter being higher than *P_NORMAL*, so a transition with this priority will fire before a transition with priority *P_NORMAL*. The priority of a transition is indicated in the bottom left corner of the transition, but it is usually omitted when it is *P_NORMAL*.

**Figure 5:**
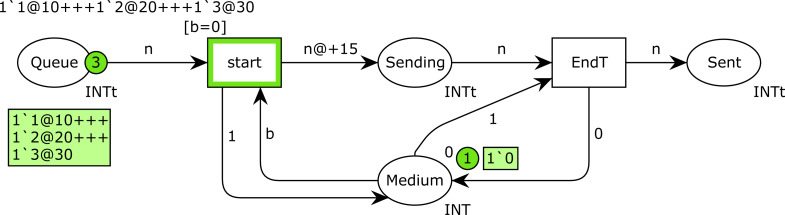
CPN modeling a simple message transmission.

**Example.** Figure 5 shows a CPN modeling a simple message transmission. In this CPN, place *Queue* has the color set *INTt* (timed integer) and its initial marking is indicated above the place. It initially contains three timed tokens, with values 1, 2 and 3, which, respectively, are available at times 10, 20 and 30. These tokens represent 3 messages to be sent, with values 1, 2 and 3. Their timestamps are the times at which these messages can be sent, but only if the medium is free. Place *Medium* has the color set *INT*, and it only contains one token, whose value can be 1 (busy) or 0 (free). It is initialized to 0 (medium free). In CPN Tools, as the simulation is performed, the current marking in every place is represented in a green box beside the place and the current number of tokens in a green circle. Thus, Fig. 5 shows the initial marking of this CPN, before firing any transition.

Transition *start* has a guard [b =0], which means that it can only be fired when the token in place *Medium* is 0 (medium free). Thus, transition *start* fires at time 10, and as a consequence, the token 1‘1@10 is removed from place *Queue*, a new token *1‘1@25* is produced in place *Sending*, and the medium state is changed to 1 (busy). Note the delay of 15 time units for the new token produced in place *Sending*. This token represents the message transmission, which will finish at time 25. Transition *EndT* (end of transmission) is then fired at time 25, which removes the token from place *Sending*, and changes the medium state to 0 again. The firing of *EndT* also produces a token in place *Sent* (messages that have been sent). Afterwards, the transmission of the message with value 2 starts at time 25, and its transmission terminates at time 40. Finally, the last message is sent at time 40, and its transmission terminates at time 55.

The example shown in Fig. 5 is very simple. In general, CPNs can be very large, with a considerable number of places, transitions and arcs. In these cases, we use the hierarchical features of CPN Tools. In particular, in this article, we use CPN pages, which allow us to split a large CPN model into several pages, which contain the different parts of the same CPN model, where the *glue* are the so-called *fusion places*, which are sets of places used on different pages, but that correspond to the same place from a formal viewpoint. These places are identified as part of the same fusion place by a blue label in the bottom left part of the place. Those places with the same label belong to the same set of fusion places, and are functionally equivalent.

## CPN Model

In this section, we present the CPN model for the IEEE 802.11e protocol with station mobility. This model extends the 802.11e CPN model presented in our previous article (Coronado et al., 2021). This new CPN consists of ten pages, two pages more than the version in Coronado et al. (2021), and the structure is different since the CPN model has been modified to include station mobility and take the visibility groups into account when the stations are transmitting messages.

**Figure 6:**
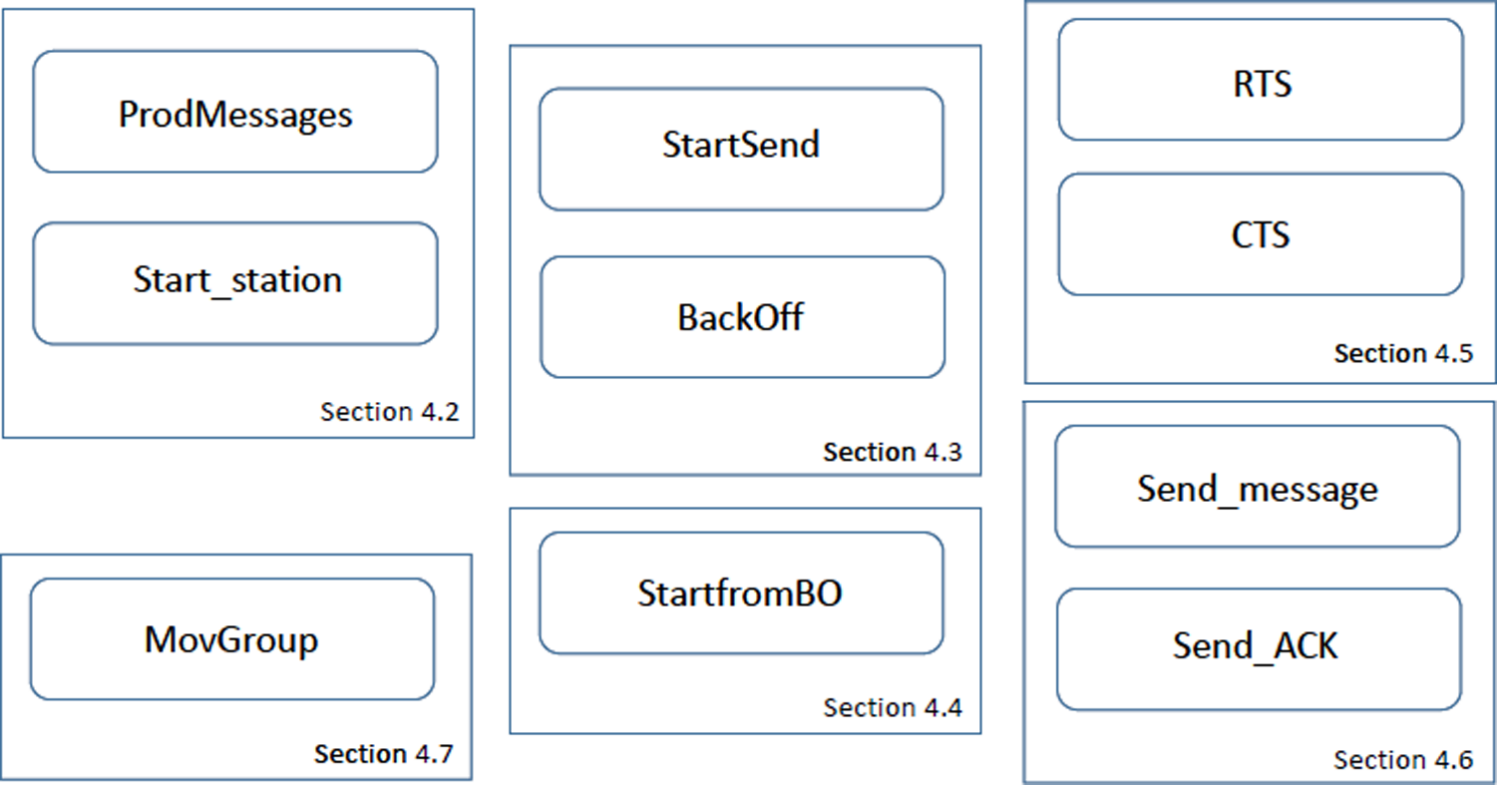
The CPN model’s pages.

Figure 6 shows the overall CPN model structure, indicating the sections in the article in which these CPN pages are described. The ten pages have been organized into six blocks. This structure allows us to add further functionalities and enable/disable some protocol features. The different blocks can be briefly described as follows:

• The first block consists of the *ProdMessages* and *Start_station*. The first page defines the traffic statistics, *i.e.,* the message arrival pattern for each traffic priority. In the current version, two traffic types have been defined, namely greedy and intermittent. The latter follows an exponential distribution, but other distributions could easily be considered. On the *Start_station* page, the initial part of the sensing process of the DCF protocol is implemented.
• The *StartSend* and *Backoff* CPN pages implement the main part of the sending process. In *StartSend* we distinguish whether RTC/CTS is applied or not. The *Backoff* page only becomes active when a Backoff process is required, before sending a message.
• The third block only consists of the *StartfromBO* page, whose purpose is to monitor the channel status, and freeze or decrement the backoff counter accordingly.
• The block consisting of the *RTS* and *CTS* pages implements the RTS/CTS protocol, as defined by the IEEE 802.11 standards. Depending on the value of the constant *RTS* in the CPN model (1 or 0), this block will be enabled or disabled. In our study, we have evaluated the network performance in both scenarios, *i.e.,* with and without the RTS/CTS protocol.
• The *Send_message* and *Send_ACK* pages implement the handshaking process as defined by the protocol. Depending on the outcome, a successful communication or a collision may arise.
• Finally, the *MovGroup* page implements the mobility process. This module is specific to the model of mobility under study. In our case, we have considered two disjoint coverage groups, with the stations in group 2 moving towards group 1 in a deterministic way.

There are many changes with respect to the version presented in Coronado et al. (2021). Page *ProdMessages* is nearly the same, but the other CPN pages have been modified in order to include the new features considered in this article. Thus, the color set of some places has changed, mainly to include mobility, and therefore the arc inscriptions and the functions used in them. Furthermore, the structure of some pages has changed to include mobility; and to improve readability, some pages have been split into two pages, which is the case with the Backoff page and the RTS/CTS control.

### Model configuration

We first present the parameters that allow us to configure the CPN model for the 802.11e protocol with station mobility (see Table 2). Some of them will not change in the course of the experiments, and the specific values considered for these are shown in Table 3. Type 1 stations generate saturated traffic, so they immediately produce a new frame after successful transmission. Type 2 stations use an exponential distribution whose rate can be assigned depending on the type of traffic (*r*2). The variables *n1p* and *n2p* indicate the number of type-1 and type-2 stations for each type of traffic: background, best effort, video and voice.

### ProdMessages and Start_station CPN pages

**Figure 7:**
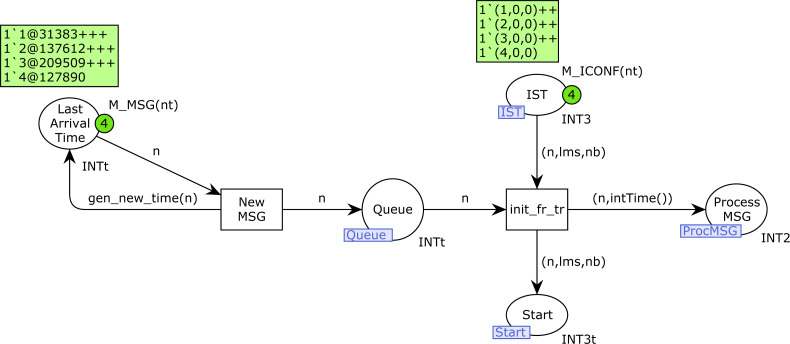
ProdMessages CPN page.

Figure 7 shows the *ProdMessages* CPN page. The CPN depicted was configured with 4 stations of type 2, with 2 sending Background traffic and 2 Voice traffic, *i.e., n*2*p* = (2, 0, 0, 2) and *n*1*p* = (0, 0, 0, 0).

The transition *NewMSG* is used to produce new messages and *init_fr_tr* to take a frame from the queue and start transmission. The starting time for every transmission is saved in the place *Process_MSG*. The place *IST* is used to activate new transmissions from each station. A token (*n*, *lms*, *nb*) in this place indicates that station *n* is ready to start a new transmission, where *lms* is the last medium state known by this station (1 if busy, 0 otherwise), and *nb* indicates whether this station requires a Backoff algorithm to be executed before the transmission.

**Figure 8:**
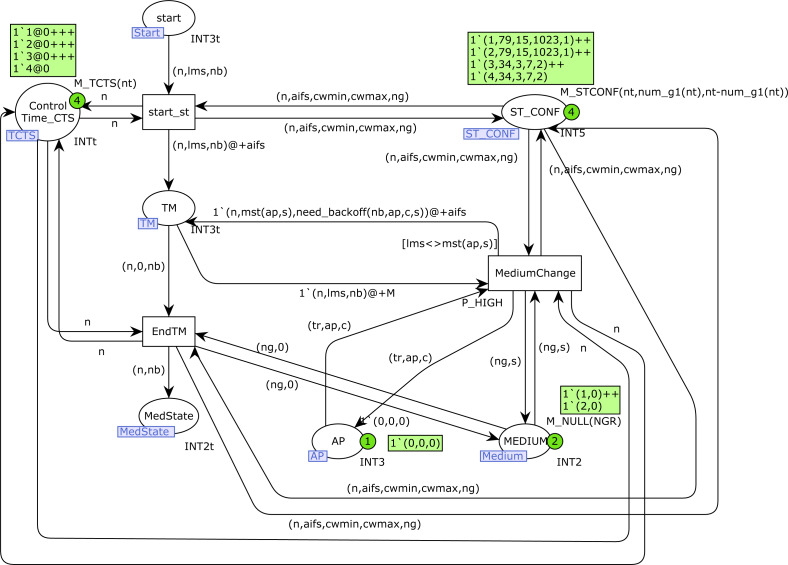
Start station CPN page.

The *Start_station* CPN page is shown in Fig. 8. Place *MEDIUM* indicates the current medium state, taking into account the visibility groups. For each visibility group it contains the number of stations that are currently transmitting in that group, so the marking consists of pairs (*ng*, *s*), where *ng* is a group number and *s* the number of transmissions in that group. The information regarding the access point (AP) is indicated by the color set of place *AP* : current number of incoming transmissions, AP transmitting(1 or 0, as a Boolean), and collision detected (1 or 0). The color set of *ST_CONF* contains the following information: station number, AIFS, CWMIN, CWMAX and group number. AIFS, CWMIN and CWMAX are assigned using the priority level of each station, and the group number is initialized randomly, but with the goal of having the same number of stations in each group, or one station more in group 1 when the total number of them is odd.

**Table 2:** CPN model configuration parameters.

**Name**	**Type**	**Description**
RTS	int	1 if RTS/CTS used, 0 otherwise
nt1	int	#stations type 1 (saturated traffic)
nt2	int	#stations type 2 (intermittent traffic)
r2	(real,real,real,real)	Exponential rates for type 2 stations
n1p	(int,int,int,int)	#stations of type 1 by priorities
n2p	(int,int,int,int)	#stations of type 2 by priorities
CWMIN	(int,int,int,int)	CWMIN values by priority
CWMAX	(int,int,int,int)	CWMAX values by priority
AIFS	(int,int,int,int)	AIFS values by priority (microseconds)
Payload	(int,int,int,int)	Payload values by priority
DataRate	(int,int,int,int)	Data rate values by priority
SIFS	int	SIFS value
SlotTime	int	Slot times

**Table 3:** Specific parameters.

Name	Value
r2	(1.0/10000.0, 1.0/10000.0, 1.0/10000.0, 1.0/10000.0)
CWMIN	(15, 7, 3, 3)
CWMAX	(1023, 1023, 15, 7)
AIFS	(79, 43, 34, 34)
SIFS	16
SlotTime	9

When a station *n* is ready to start transmission, the *start* place is marked with one token (*n*, *lms*, *nb*). Transition *start_st* starts the medium check, but it can be delayed if RTS/CTS is used and a CTS message has been received, which makes this station wait (control by place *Control_Time_CTS*). A medium change is detected by the transition *MediumChange*, which is only fired when the current medium state is different from *lms*. The function *mst* is used to capture the current medium state, and *need_backoff* determines whether a Backoff should be performed or not. We omit here the details of these functions, which can be found in Appendix A. When the medium is free for this visibility group, transition *EndTM* is fired and the station *n* can start the transmission (place *MedState* is marked with a token (*n*, *nb*), where *nb* indicates whether Backoff is required).

### StartSend and Backoff CPN pages

**Figure 9:**
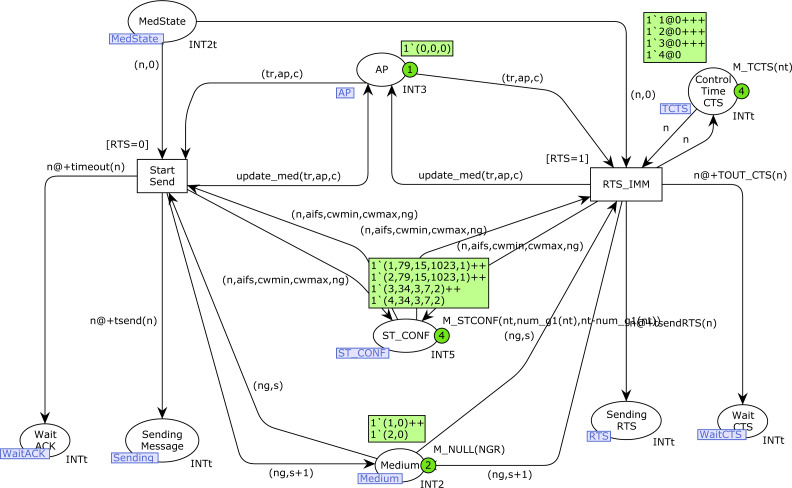
StartSend CPN page.

Figure 9 shows the *StartSend* CPN page. Transition *Start_Send* is used when RTS/CTS is not used (parameter *RTS* is 0) to start a message transmission, whereas *RTS_IMM* is used when RTS/CTS is used to start the RTS transmission. These transitions update the medium state information in the place *Medium*, as well as the *AP* state information in the place *AP*. In the event of a collision, this is indicated in the third component of the token in *AP*. Both transitions are loop-connected^2^ 2There is an arc from the place to the transition and vice versa, with the same inscription in both arcs.to the place *ST_CONF* to obtain the binding for the *ng* variable (current number of the visibility group of station *n*). When these transitions are fired, a new token is produced in the corresponding *Sending* place (*Sending_Message* or *Sending_RTS*, depending on the use of the RTS/CTS protocol), as well as a token in the corresponding *Wait* place. Both the *Wait_ACK* and *Wait_CTS* places are used to set a time-out and thus detect a failure in transmission.

**Figure 10:**
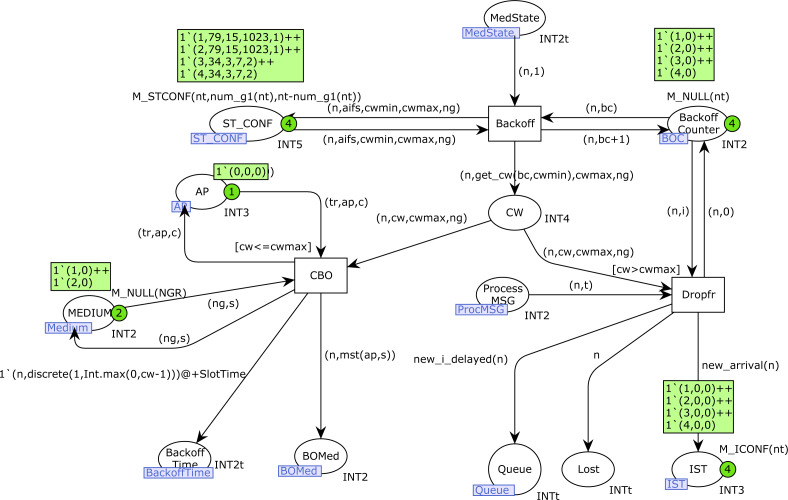
Backoff CPN page.

The first part of the Backoff algorithm is implemented on the *Backoff* CPN page, as shown in Fig. 10. The transition *Backoff* starts the algorithm and increases the Backoff counter for station *n* in the place *Backoff_Counter*. The current value of CW for station *n* is obtained with the function *get_cw*, and it is stored in the place *CW*, together with the CWMAX value and the current number of the visibility group of the station *n*. When the current value of CW is greater than CWMAX, the transition *Dropfr* is fired, which means that a frame is lost. Lost frames are indicated by the marking of the place *Lost*. In the case of stations of type 1, which produce saturated traffic, a new frame is produced in the place *Queue* for this station, with the goal of starting a new transmission. A new token is also produced in the place *IST*, in order to activate a new transmission for this station *n*. In this case, Backoff is only required for type-1 stations(function *new_arrival*). The firing of *Dropfr* also removes the token that was produced for this transmission in the place *Process_MSG*.

Otherwise, when CW is less than or equal to *CWMAX*, the transition *CBO* is fired, which assigns the Backoff counter (place *Backoff_Time*) as indicated in the section “IEEE 802.11-Based Wireless Networks”, and creates a new token for this station *n* in the *BOMed* place, which contains the current medium state known for this station during a Backoff (function *mst*).

### StartfromBO CPN page

**Figure 11:**
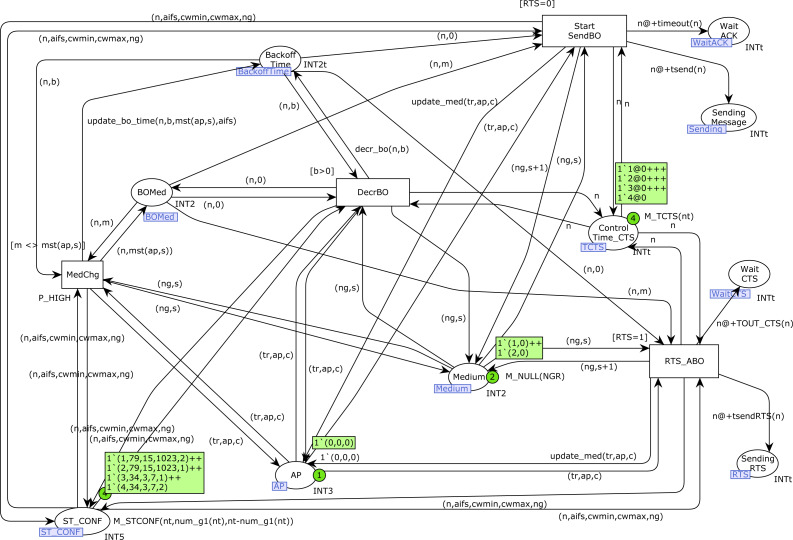
StartfromBO CPN page.

Figure 11 shows the *StartfromBO* CPN page, which corresponds to the second part of the Backoff algorithm, in which the Backoff counter (place *Backoff_Time*) is decremented until reaching 0. Note that we can have several stations in Backoff at the same time, *i.e.,* there can be several tokens in the place *Backoff_Time* corresponding to different stations.

Once the Backoff counter is 0, either a frame or RTS transmission starts, depending on the value of the constant *RTS*. However, the Backoff decrement must stop when the medium is busy, and the visibility groups must be taken into account when updating this information for each station. The transition *DecrBO* fires at the end of each slot time when the medium is free for station *n*. This transition decrements the Backoff counter in the place *Backoff_Time*. When this value is 0, either transition *Start_SendBO* (start frame transmission, when RTS/CTS is not used) or *RTS_ABO* (start RTS transmission, when RTS/CTS is used) is fired. In addition, when there is a medium state change, the transition *MedChg* is immediately fired (note the high priority) to update the information in the place *BOMed* and update the time at which the token in the *Backoff_Time* for station *n* will be available. Specifically, when the medium becomes free, a delay of AIFS microseconds must be applied (function *update_bo_time*), because the medium must be free during an AIFS period to restart the Backoff decrement.

Transitions *Start_SendBO* and *RTS_ABO* initiate a transmission, so a token is produced in the corresponding *Sending* place, as well as in the corresponding *Wait* place, as we showed on the *StartSend* CPN page, when a Backoff process was not required.

### RTS and CTS CPN pages

**Figure 12:**
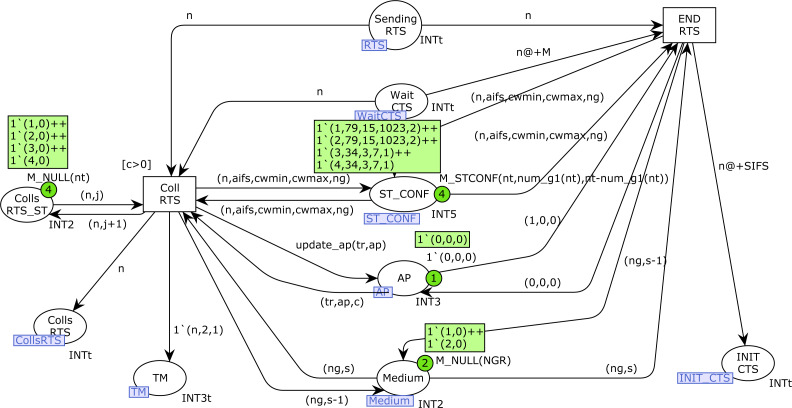
RTS CPN page.

**Figure 13:**
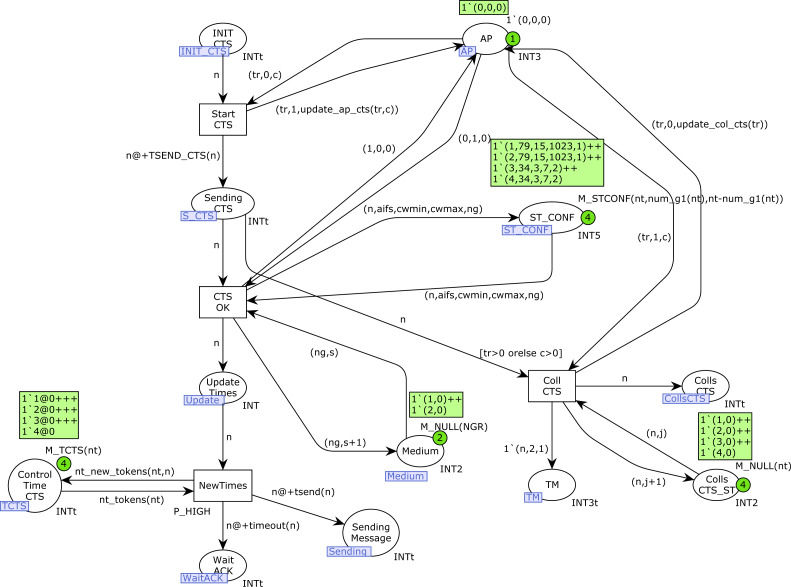
CTS CPN page.

Figures 12 and 13 show the *RTS* and *CTS* pages of the model. The RTS page is activated when a station is sending an RTS frame(place *Sending_RTS* marked). Obviously, several stations could try to send their RTS frames simultaneously, so there can be several tokens in this place. Collisions are detected by the marking in the *AP* place (third component). Thus, *END_RTS* is fired when there have not been any collisions in the transmission of an RTS frame, removing the time-out token in the place *Wait_CTS*. Otherwise, when there has been a collision, once this time-out expires the transition *Col_RTS* is fired, which records the collision in both the *Colls_RTS_ST* (counter of collisions for each station) and *Colls_RTS* (all collisions) places. The token written in the place *TM* allows the station to retry the transmission, but it must first check the medium state again, and Backoff is required (see Fig. 8: Start_station CPN page).

The firing of *END_RTS* writes a token in the place *INIT_CTS*, which, after the SIFS time, starts the CTS page (Fig. 13) for station *n*. Transition *Start_CTS* is used to send the CTS message, which updates the *AP* state information. Collisions of CTS frames occur when a station starts an RTS transmission just before the AP starts the CTS transmission. In this case, the transition *Coll_CTS* is fired, which records the collision in both the *Colls_CTS* (all collisions) and *Colls_CTS_ST* (collisions by station) places. Otherwise, when the CTS transmission is completed(firing of transition *CTS_OK*), the control times of the tokens in the *Control_Time_CTS* place must be updated for all the stations except that one sending the message. It should be remembered that these tokens are used to stop the station transmission activity when a CTS has been received. This action is performed by the firing of the transition *NewTimes*, which also writes a token in the *Sending_Message* place to indicate the transmission, and another one in the *Wait_ACK* place to detect failures.

### SendMessage and SendACK CPN pages

**Figure 14:**
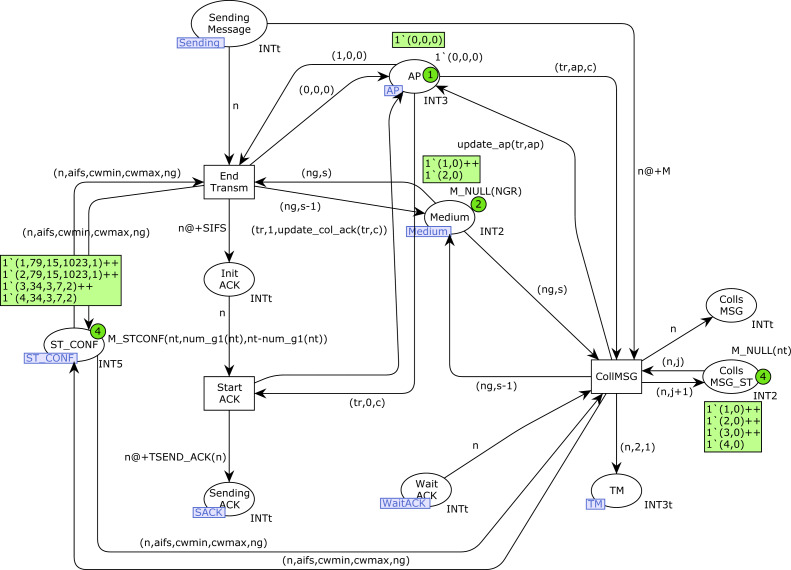
SendMessage CPN page.

Figure 14 shows the *SendMessage* CPN page. When a station *n* is sending a message there will be a token in the place *Sending_Message* with its station number. If no collision has occurred, this transmission finishes correctly with the firing of the transition *End_Transm*. Otherwise, in the presence of collisions, the transition *CollMSG* will be fired, indicating the collision in both the *Colls_MSG* and *Colls_MSG_ST* places. In this case, a new token (*n*, 2, 1) is also produced in the *TM* place, in order to retry the transmission, but the medium state must be checked and a Backoff process is required (see Fig. 8). After a successful transmission, the ACK transmission starts (place *Sending_ACK* marked by the firing of transition *Start_ACK*).

**Figure 15:**
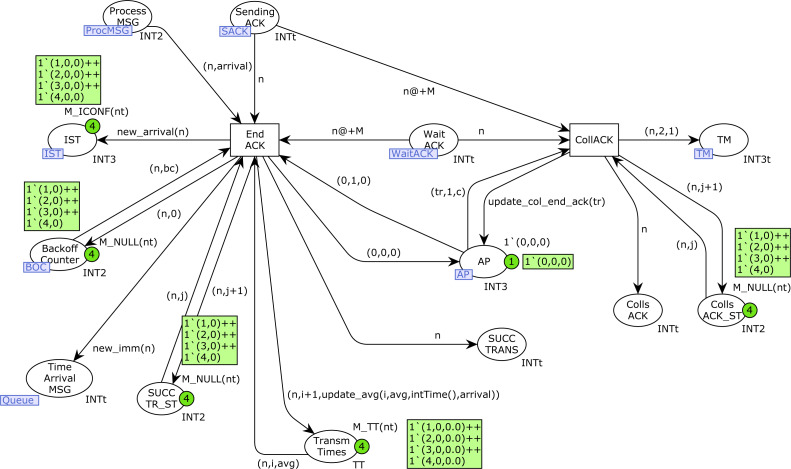
SendACK CPN page.

In Fig. 15 we can see that an ACK transmission can terminate correctly (transition *End_ACK*) or there can be an ACK collision (transition *CollACK*). ACK collisions are recorded in both the *Colls_ACK* and *Colls_ACK_ST* places, and a new token is produced in the *TMP* place to retry the transmission. Successful transmissions are recorded in the *SUCC_TRANS* and *SUCC_TR_ST* places. In addition, the number of successful transmissions for each station and the average transmission times are calculated in the place *Trans_Times*. Moreover, in the case of type-1 stations, a new token is produced in the place *Time_Arrival_MSG* to start a new transmission (function *new_imm*).

### MovGroup CPN page

We use a deterministic model of mobility, with several goals in mind. First, we consider specific mobility scenarios that we can analyze, and the results obtained from simulations can be interpreted in relation to those scenarios. In addition, the simulations must be repeated several times in order to obtain representative results from a statistical point of view, so these executions must be repeated on the same mobility assumptions.

The simulations are executed for a total model time of 15 s, with periods of mobility every 3 s. The total simulation time was chosen to be long enough in order to allow us to obtain performance results from which we can draw conclusions regarding the impact of the network configuration, the number of stations and their mobility. The time for the mobility windows (3 s) was also chosen to allow us to see the impact on performance of the current number of stations in each group. As an illustration, for a configuration with a single type-1 station producing voice traffic, *i.e., n*1*p* = (0, 0, 0, 1), *n*2*p* = (0, 0, 0, 0), we obtain approximately 11561 messages sent in 3 s using RTS/CTS, and 17910 without RTS/CTS. When RTS/CTS is used, the transmission time for a single message is approximately 259 microseconds (Backoff included). These numbers justify the decision of taking 3000000 microseconds (3 s) for each period, which is enough time to obtain reliable performance results for each period. Although the residence time of the stations in a given coverage sector will heavily depend on the scenario, such as pedestrians on the move in a building or outdoors or vehicular communications, our results allow us to evaluate the performance of the MAC protocol mechanisms as the network setup changes.

Stations are initially divided into two mobility groups of the same size, unless there is an odd number of stations, in which case there will be one more station in group 1. Then, every 3 s some stations move from group 2 to group 1, thus modeling a situation in which a group of people tend to converge at the same location, and thus get closer to each other. However, mobility is problematic when a station is trying to send a frame, so to avoid problems we restrict mobility, and stations can only move to group 1 when they are not transmitting. As a consequence, mobility is delayed until the corresponding station is not transmitting a frame. This is a small delay that hardly affects the comparability of the results when simulations are repeated several times.

In general, for a total number of *n* stations, when *n* is even, there are initially *n*/2 stations in each group, and when *n* is odd, there are (*n* + 1)/2 stations in group 1 and (*n* − 1)/2 in group 2. Mobility is permitted at times 3, 6, 9 and 12 (4 cycles), and the number of stations that move at cycle *i* is computed as follows:

(1) \begin{eqnarray*}\mathit{St_to_move}(m,i)= \left\{ \begin{array}{@{}ll@{}} \displaystyle M[m,i]&\displaystyle \mathit{if}m\leq 4\\ \displaystyle M[1+((m-1)\,\mathit{mod}\,4),i]+(m-1)/4&\displaystyle \mathit{otherwise} \end{array} \right. \end{eqnarray*}

where/denotes the integer division and *m* is the number of stations that are initially in group 2, *i.e.,* the total number of stations that must move to group 1 in the 4 cycles of mobility. Function *M* is defined as indicated in Table 4.

For instance, for *n* = 3 (total number of stations), there are initially 2 stations in group 1 and 1 station in group 2. Only one station must move to group 1 (*m* = 1), and this movement occurs at cycle 3 (time 9). In the case where *n* = 16, we have *m* = 8, so 2 stations move to group 1 at each cycle.

**Figure 16:**
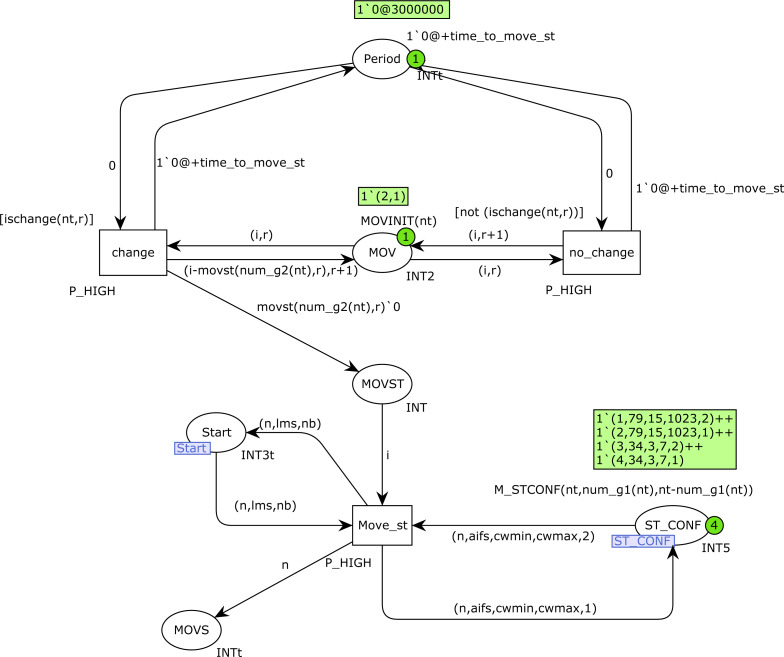
MovGroup CPN page.

In Fig. 16, the place *PERIOD* indicates the following time at which the movements will take place. It is updated with either the firing of transition *change* or *no_change*, which increment the token timestamp with the constant *time_to_move_st* (3000000). Place *MOV* indicates the stations to move at a certain cycle. Transition *no_change* is fired when no movement must be made at the current cycle, and thus it simply increments the second value in the token in *MOV* (current cycle). Otherwise, when one or several movements must be made, transition *change* is fired, which updates *MOV* and produces one token for every movement in the place *MOVST*. Afterwards, transition *Move_st* fires (due to its high priority) as many times as the tokens we have in *MOVST*, and at each firing it changes the visibility group of one station that is in the *Start* state from 2 to 1. Note that this restriction can slightly delay the movements of some stations, until they are in the *Start* state (once they have finished some transmission), but as mentioned above, these little delays hardly affect the final results.

**Table 4:** M. station mobility at each cycle for *n* ≤ 4.

Total	Cycle			
Movs	1	2	3	4
1	0	0	1	0
2	0	1	0	1
3	0	1	1	1
4	1	1	1	1

### Other models of mobility

The CPN model presented is designed to support any number of visibility groups, the only page that needs to be changed is the *MovGroup* CPN page, which implements the specific model of mobility considered. Function *M_STCONF*, which produces the initial marking of place *ST_CONF*, should also be extended to indicate the initial number of stations in each visibility group.

On the *MovGroup* CPN page the changes would mainly affect the bottom part of the CPN, from place *MOVT* downwards. In principle, both functions *ischange* and *movst* should be rewritten according to the new model of mobility, but these changes do not affect the net structure. However, depending on the model of mobility considered, the way in which mobility is implemented by transition *Move_st* could require structural changes or just simple modifications in the arcs connecting *Move_st* with place *ST_CONF*.

### Model validation

Validation was carried out by defining a set of test cases which captured the protocol requirements, and all of them were checked by establishing the appropriate scenarios that allowed their validation. Table 5 shows these test cases.

**Table 5:** Validation: test cases.

#	Test Case	Page
1	Messages are produced correctly.	ProdMessages
2	Transmission starts after medium idle for AIFS µs.	Start_station
3	Backoff required if medium found busy	Start_station.
4	Station *n* starts message transmission when *MedState* contains one token (*n*, 0) and *RTS* = 0.	StartSend
5	Station *n* starts RTS transmission when *MedState* contains one token (*n*, 0) and *RTS* = 1.	StartSend
6	Station *n* starts Backoff when *MedState* contains one token (*n*, 1). Content window *cw* generated correctly and frame lost if *cw* > *CWMAX*.	Backoff
7	Decrement of Backoff counter is only applied when station not affected by CTS control time and medium not busy for AIFS µs. Change of medium state detected by transition *Medchg*.	StartfromBO
8	Station *n* starts message transmission when *RTS* = 0, token (*n*, 0) in place *Backoff_Time* and station *n* not affected by CTS control time.	StartfromBO
9	Station *n* starts RTS transmission when *RTS* = 1, token (*n*, 0) in place *Backoff_Time* and station *n* not affected by CTS control time.	StartfromBO
10	RTS transmission terminates for station *n* after *tsendRTS(n)* µs if no collision occurred.	RTS
11	RTS collision detected for station *n* when other station or the AP sending. Station *n* retries transmission with Backoff activated.	RTS
12	CTS transmission starts after SIFS µs, after correct RTS transmission for station *n*.	RTS
13	A CTS collision occurs when a station starts RTS transmission and AP ready to send CTS.	CTS
14	CTS terminates correctly when no collision detected.	CTS
15	Message transmission terminates correctly for station *n* in *tsend(n)* µs when no collision detected.	SendMessage
16	Transition *CollMSG* fires in the event of a message collision.	SendMessage
17	After a successful transmission the ACK transmission starts after SIFS µs.	SendMessage
18	ACK transmission terminates correctly after *TSEND_ACK(n)* µs when no collision detected.	SendACK
19	Transition *CollACK* fires in the event of an ACK collision.	SendACK
20	Model of mobility is correct.	MovGroup

## Evaluation

This section describes the performance evaluation carried out in this work, taking as reference the model presented in “CPN Model”. The evaluation was based on an IEEE 802.11 network, with the ability to handle different QoS traffic priorities(as defined by the IEEE 802.11e amendment). In this regard, each traffic type is characterized by a distinct transmission rate and packet size. The physical layer used as a reference to model the transmission rate corresponds to IEEE 802.11ac (at 5 GHz). Moreover, the evaluation covers the ability of the network to switch the RTS/CTS protocol on and off. Since our goal is to evaluate the performance of the MAC protocol in a mobility scenario, we have considered a BS covering an area where different groups of stations are distributed over two coverage areas. This scenario allows us to evaluate the RTS/CTS mechanism forming part of the MAC layer under a varying number of stations with different patterns and priorities. One of our previous works focused on the performance of the MAC priority mechanism of the IEEE802.11 standard in an extreme case, namely one in which none of the stations was able to detect the presence of any other station (Coronado et al., 2021). The scenario here represents a more realistic case, where stations will move from one sector to another covered by a BS implementing the RTS/CTS as a means to mitigate the hidden-node problem. Our results include a comparative evaluation of the performance when the RTS/CTS is disabled.

We aim to evaluate the effect of user mobility, as well as analyze the impact of collision zones in this case. As mentioned above, the mobility of the stations directly impacts the visibility that the stations have among each other, even if they are connected to the same AP. For that reason, the evaluation covers the same tests considering the case when the RTS/CTS protocol is disabled or enabled in order to assess the performance boost provided by this protocol in scenarios where a big group of users are moving around but suffer from constant collisions. In addition, the proposed scenarios cover the behavior of various traffic types as defined by the IEEE 802.11e amendment. Throughout our study, the simulations were performed using traffic with the highest priority (voice), the lowest priority (background), and a mix of both priorities (voice and background). The channel access parameters employed by these two traffic types are different, providing faster, but also more sensitive access to the VO traffic compared with BK traffic. The remaining parameters used to configure the various scenarios are listed in Table 6. Note that both the payload and the datarate are configurable for each traffic type, while the remaining parameters are generic for the network itself.

**Table 6:** Main parameters defining the IEEE 802.11 network evaluated.

Parameter	Traffic type	Value
Payload	Background	1,000 bytes
	Voice	170 bytes
Datarate	Background	65 Mbps
	Voice	65 Mbps
Basic datarate	–	6 Mbps
ACK size	–	20 bytes
RTS size	–	14 bytes
CTS size	–	14 bytes
PHY header	–	24 bytes
MAC	–	34 bytes
Slot time	–	9 µs
SIFS	–	16 µs

For each scenario, ten simulations were executed using the simulation replication capabilities of CPN Tools, which allow us to automatically obtain the performance results and collect statistically reliable data. In this regard, all the figures presented represent the average of the results obtained in the ten rounds, together with the associated 95% confidence interval (CI). It is important to highlight that due to the small variance obtained over the rounds of the experiments, the CI is almost visually imperceptible in some plots. These small variances demonstrate that ten simulations are enough to provide reliable results for all the experiments. Regarding the simulation times and mobility pattern, they were set by taking into account that our main goal was to evaluate the impact that the number of stations, priority classes and the use of RTS/CTS have on the performance of the network protocol. By setting the residence time to a fix-value of 3*s* for each observation period, it should be possible to evaluate the impact of the main protocol parameters, namely number and classes of the stations and the use of RTS/CTS, on the protocol’s performance. From the analysis of the results, we should be able to evaluate the effectiveness of the protocol mechanisms and make recommendations on their use under various network operating conditions.

### Single priority scenarios

**Figure 17:**
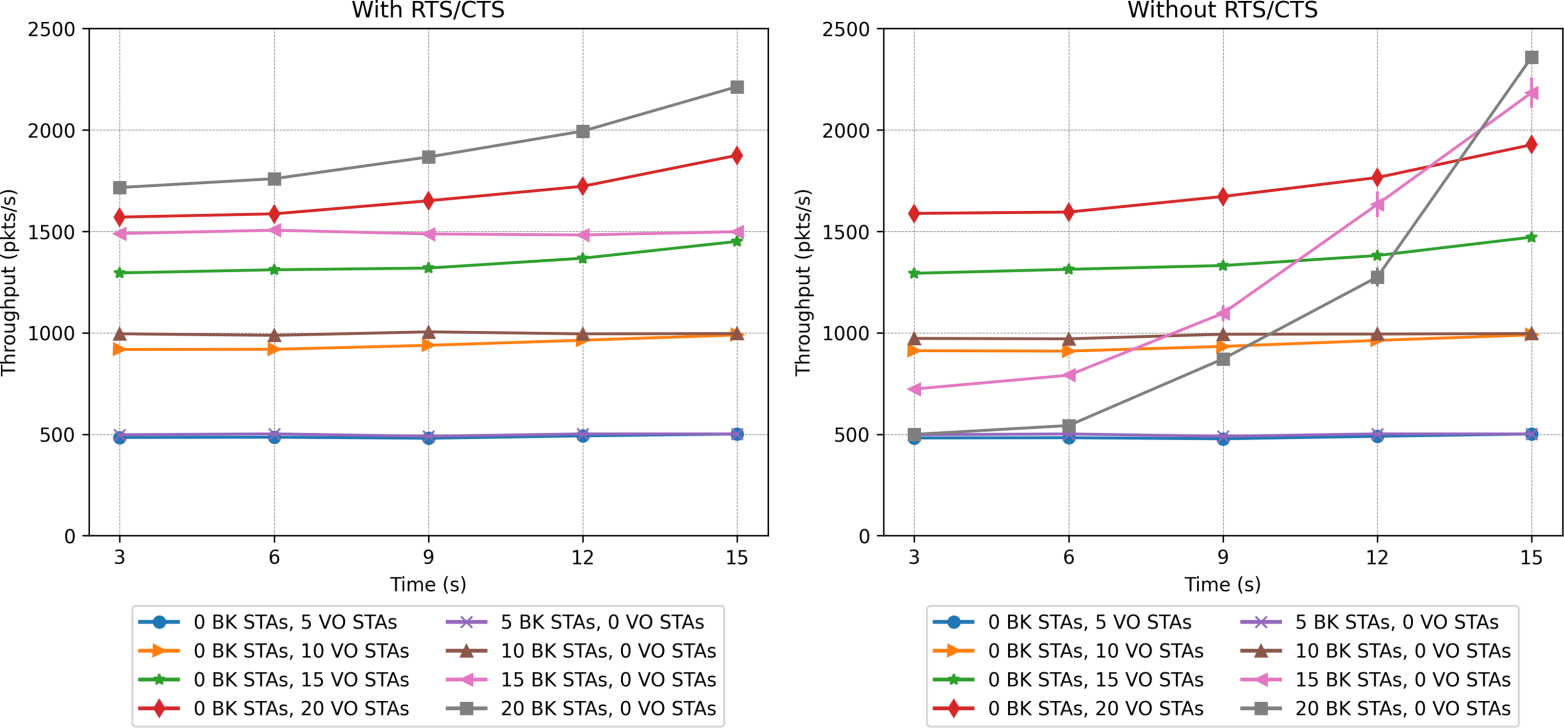
Throughput results in the mobility scenario with only one traffic type.

**Figure 18:**
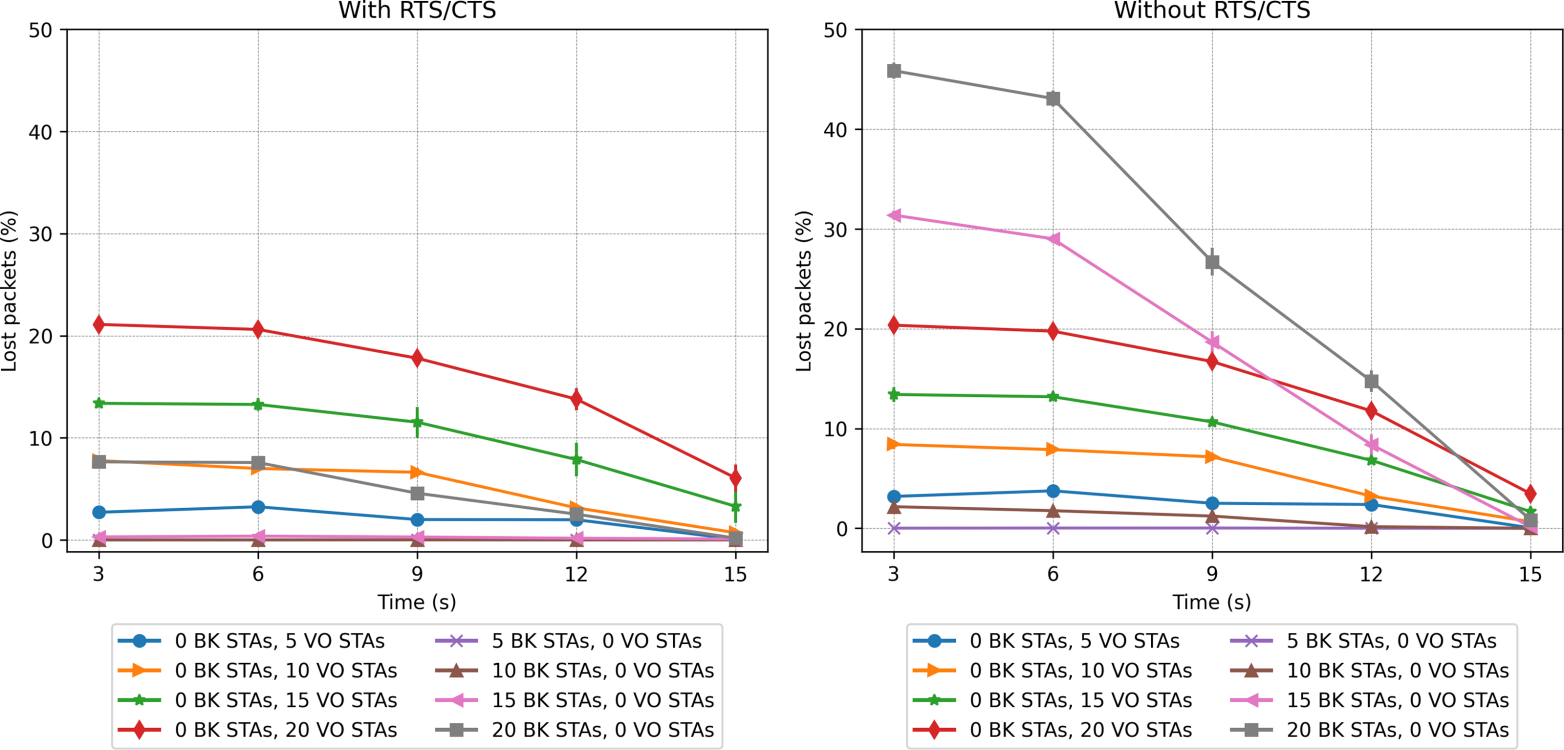
Packet loss rate in the mobility scenario with only one traffic type.

The first two scenarios explore the performance when all stations implement a single priority class, namely VO or BK. Figures 17 and 18 show the network-wide throughput and packet-loss rate results for both traffic classes. In the figures on the left, the results corresponding to the RTS/CTS protocol are presented, while the ones on the right show the results obtained without making use of the protocol. Note that in both figures, the captions in the left part of the legend correspond to the VO traffic, while the colors on the right side correspond to the BK traffic. As can be seen from the figures, the performance results of the VO are very similar in both cases, *i.e.,* with/without RTS/CTS. In the case of a network consisting of 15 and 20 VO stations, the throughput improves as the stations move into the same group. It is clear that the inability to detect the presence of a large number of active nodes has an impact on network performance. The results show that in the case of a network supporting only VO stations, the use of RTS/CTS does not help to mitigate the hidden-node problem. In fact, the IEEE802.11 standard recommends that RTS/CTS should be activated under high-load traffic conditions, with stations mobility and with packet lengths longer than a certain (RTS) threshold, typically 500 bytes.

In the case of the BK traffic, Figs. 17 and 18 show similar results for the case of 5 BK stations. When comparing the results obtained for 5 BK stations with the ones reported for five VO stations, it is interesting to note that the number of BK and VO packets transmitted during the period in which the stations are evenly distributed (time 3*s*), are quite similar. However, in Fig. 18 we can observe that the BK traffic reports a zero loss packet rate when the RTS/CTS mechanism is used, but a slight loss rate when the mechanism is disabled.Since the BK packet lengths are longer than the recommended RTS threshold, the use of the RTS/CTS mechanism proves effective. Furthermore, the BK traffic throughput is significantly higher when taking into account that the BK packet length is an order of magnitude longer than the VO packet length. In the case of a larger number of BK stations, *i.e.,* 15 and 20, Fig. 17 (left) shows that when stations are evenly distributed between the two groups, the BK throughput considerably increases with respect to the throughput reported when the RTS/CTS is disabled (Fig. 17 (right) at time 3*s*). However, the figure also shows that the use of the RTS/CTS mechanism slightly penalizes the throughput of the BK traffic in the case when all nodes are placed in the same group, *i.e.,* the throughput reported for the BK at time 15*s* is slightly higher in the case when the RTS/CTS mechanism is disabled. Figure 17 also shows that when the network’s load is high (15 and 20 stations), the throughput for BK traffic shows a much higher increase to the one reported for the VO traffic. This is the result of an increasing number of collisions of the voice traffic, given the shorter packet lengths used by the VO service. Furthermore, since the Backoff times of the voice traffic are shorter than ones implemented for the BK traffic, the VO stations attempt to access the channel at a higher rate, resulting in a higher number of collisions, and consequently a lower throughput. Note that in all cases, the deviation of the results obtained over the various repetitions of each experiment is almost negligible. For instance, we can observe one of the greatest deviations in the whole performance evaluation in Fig. 17 (right), in which for 15 BK stations at time 15s the standard deviation is 123.29. In this respect, it can be stated that this is a practically insignificant value and that no major differences can be seen over the runs of the experiments.

**Figure 19:**
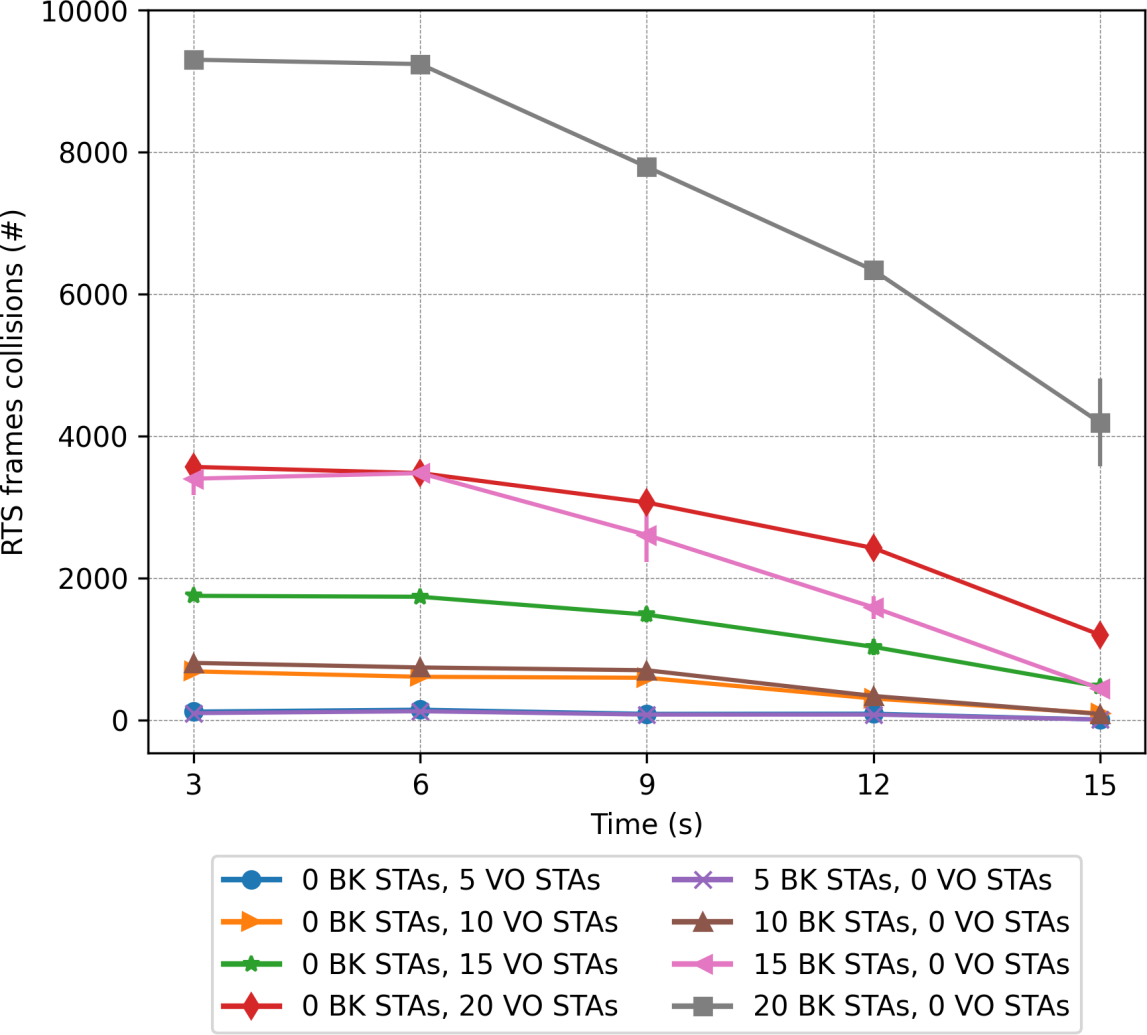
RTS collisions in the mobility scenario with only one traffic type.

A contrasting view of the above results is shown in Fig. 18, which depicts the percentage of lost packets over time. In this case, we can observe that the BK traffic is the most affected in the scenarios consisting of a larger number of stations and in the absence of the RTS/CTS protocol when the stations are evenly distributed between the two groups. This protocol proves effective at preventing the long BK data packets from colliding (Fig. 18, left). This statement is supported by the number of RTS frames colliding as a function of time (see Fig. 19). As can be seen from the figure, a higher number of collisions involving RTS packets occur at the beginning of the simulation, when half of the stations cannot sense each other. The figure also shows that the scenarios exhibiting the worst results correspond to those consisting of a larger number of stations, *i.e.,* 20 stations.

In the case of VO traffic, Fig. 18 shows that the use of the RTS/CTS does not prove as effective as for the case of the BK traffic, *i.e.,* the lost packet rate for the VO traffic with/without RTS/CTS exhibits similar values. In fact, in the scenario consisting of 20 stations, the VO packet loss rate is just slightly lower during the first period, *i.e.,* when the VO stations are evenly distributed between the two groups. In the final period, i.e, when all VO stations join the same group, the use of the RTS/CTS protocol only slightly affects the performance of the VO traffic. In fact, the use of RTS/CTS increases the network load, resulting in a higher packet collision probability. By contrast, for BK traffic, in the scenario with 20 stations, a great difference (from 45% to 8%) is observed when enabling the RTS/CTS protocol and the stations are separated into two groups without a common coverage area (at time 3*s*).

### Two-priority scenarios

**Figure 20:**
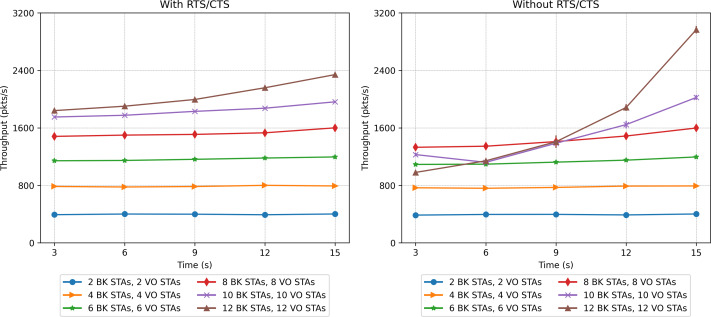
Throughput results in the mobility scenario with two traffic types: voice (sensitive) and background (insensitive).

**Figure 21:**
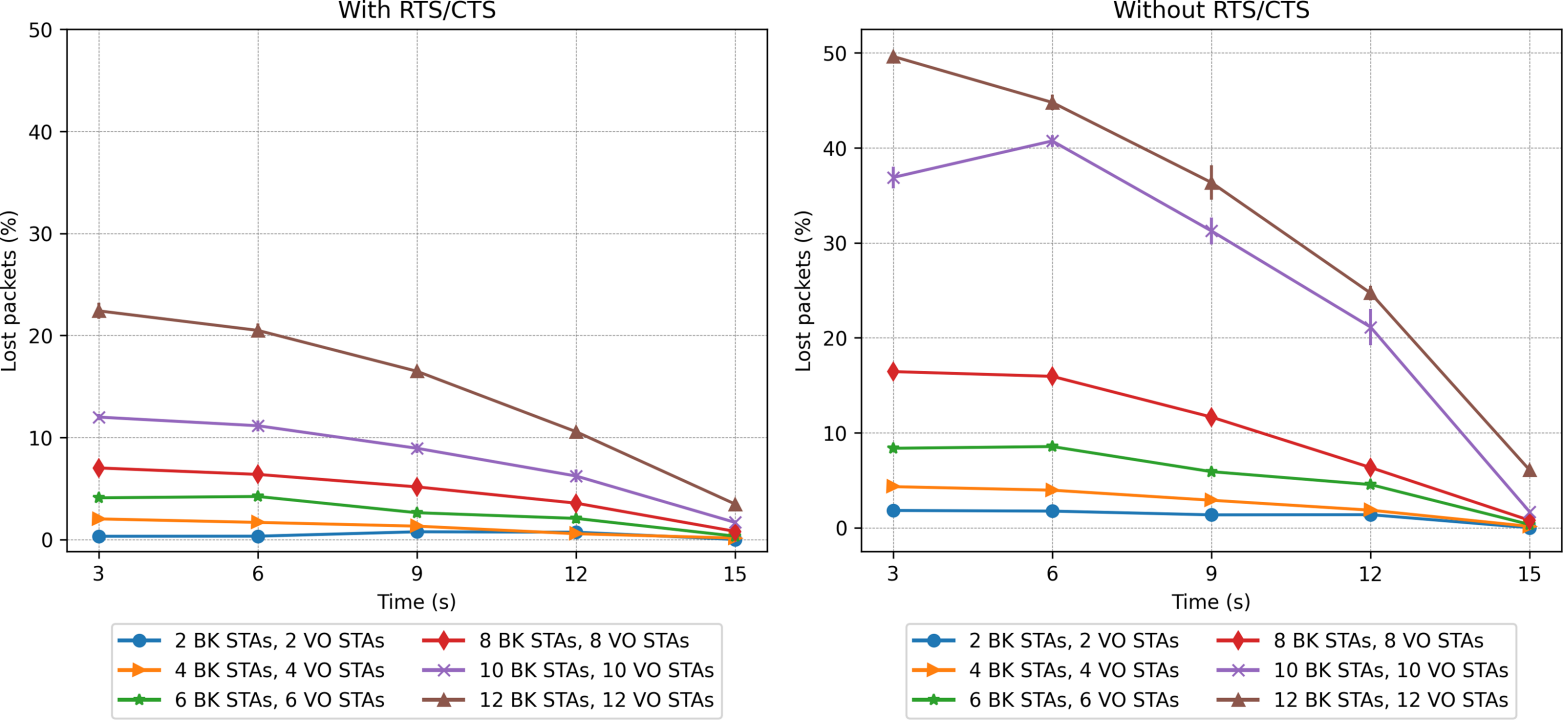
Packet loss rate in the mobility scenario with two traffic types: voice (sensitive) and background (insensitive).

The second set of scenarios considered a combined workload, with two concurrent traffic types: BK and VO. In this case, the number of stations evaluated varied from 2 to 12 for each traffic type (voice and background), which makes a range of [4, 24] in the stations present. Figures 20, 21 and 23 present the results in terms of throughput, packet loss rate and RTS collisions (when RTS/CTS is used) for the whole network. Taking into account the upper bound in the number of stations derived in the scalability analysis (24), Fig. 21 shows an extremely high percentage of messages lost when the 24 stations are evenly distributed between the two sectors. As can be observed, a protocol with such a number of messages lost would be of little use, *i.e.,* with 24 stations we are at the limits of the application of this protocol for the parameters considered.

**Figure 22:**
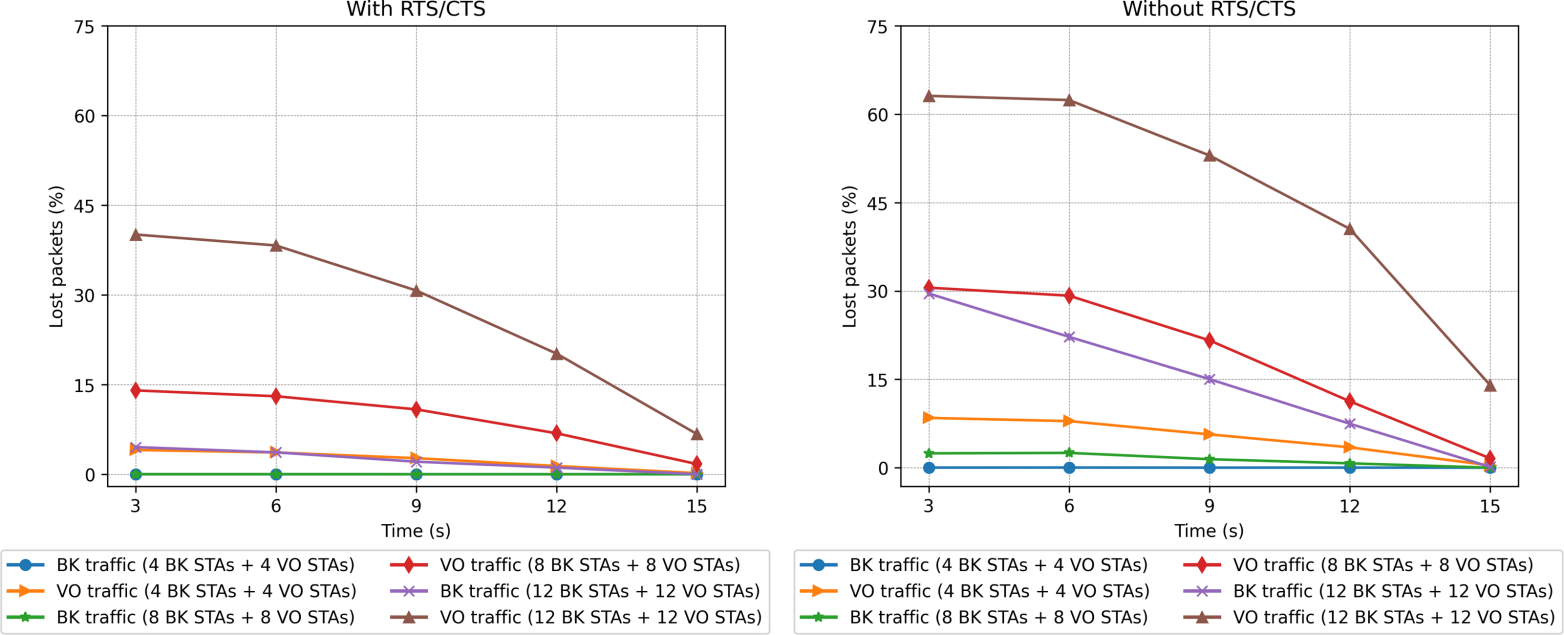
Packet loss rate (separated per traffic type) with two traffic types in the network: voice (sensitive) and background (insensitive).

**Figure 23:**
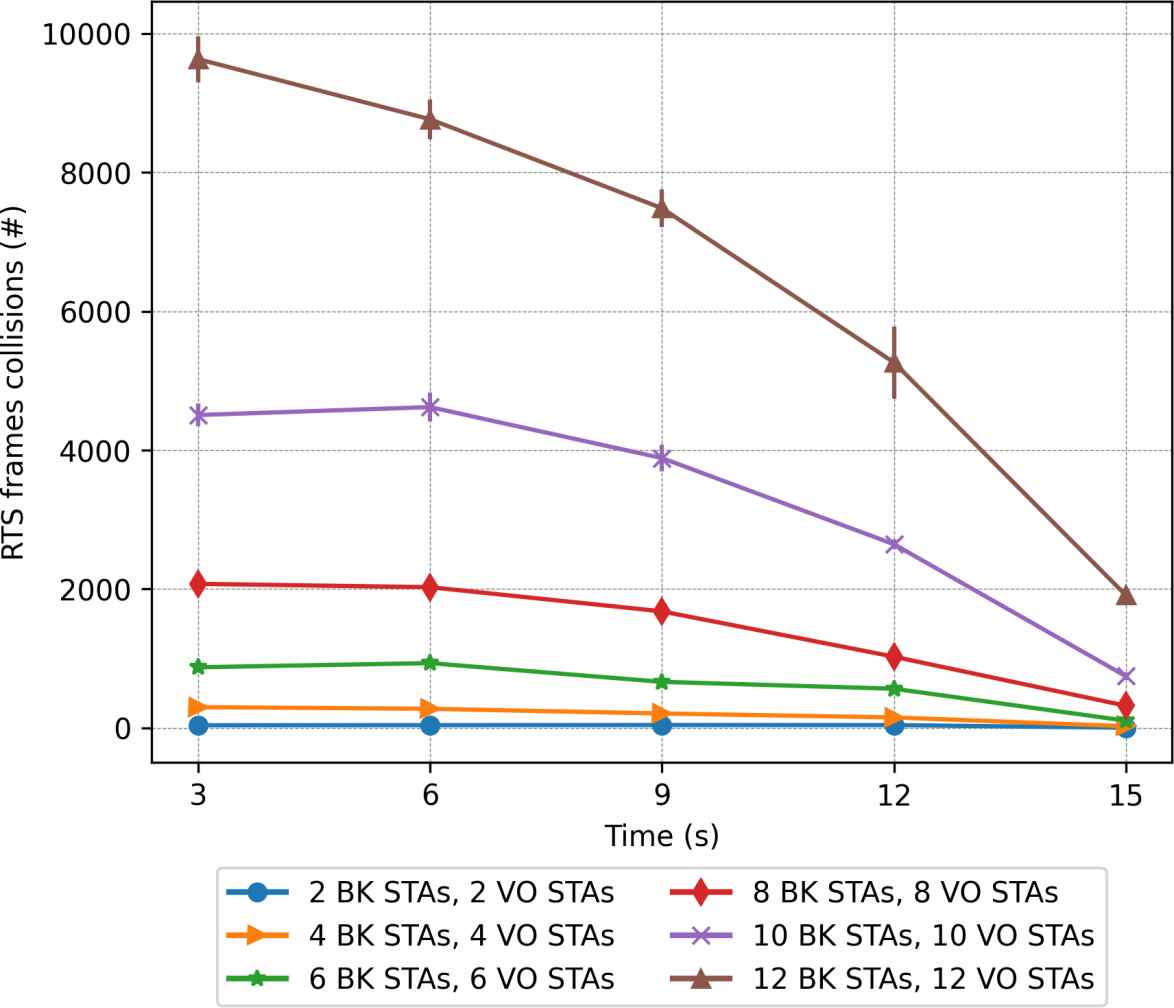
RTS collisions in the mobility scenario with two traffic types: voice (sensitive) and background (insensitive).

Figure 20 shows that the CPN model properly reflects the expected behavior for the workload considered, with both traffic types. For a low number of stations (from 2 BK + 2 VO up to 8 BK + 8 VO) the throughput is nearly the same in both cases (with and without RTS/CTS). In the case of a scenario with a larger number of stations, the use of RTS/CTS proves effective when the stations are evenly distributed between the two sectors. However, when all the stations move into the same sector (from 12 seconds on), the results when the RTS/CTS protocol is disabled are much better given that no extra control overhead is introduced in the network. It is important to highlight that under a configuration consisting of the same number of stations distributed between two traffic types, the network is exposed to a different traffic load. For instance, in the case of 10 VO and 10 BK stations, 20 in total, the network is exposed to a heavier load than in the case of 20 BK stations in our previous experiments. This is due to the shorter backoff period of the VO traffic. On the contrary, the same two-type network configuration will exhibit a lower network load when compared to a network supporting 20 VO stations.

We can also observe that the number of lost packets in the whole network is greater in all the cases in which the RTS/CTS protocol is disabled (Fig. 21). These results are confirmed by the lost packet ratio separated by traffic type, as shown in Fig. 22. Each line displays the results associated with a specific traffic type. For instance, the entry *“BK traffic (4 BK STAs + 4 VO STAs)”* shows only the percentage of lost packets corresponding to the background traffic when the network is composed of four background and four voice stations. In particular, in Fig. 22, it is possible to see that the most sensitive traffic (voice) is the one experiencing more losses given that it is more affected by collisions than background traffic. It can be observed that enabling the RTS/CTS protocol reduces the percentage of lost traffic for both types. In fact, the RTS/CTS mechanism proves more effective in improving the performance of the BK traffic when the stations are evenly distributed. For instance, in the case of 12 BK and 12VO stations, the BK packet loss drops from 30% to approximately 6%, *i.e.,* a five-fold drop. As for the VO traffic, when RTS/CTS is enabled the lost packet percentage drops by approximately one fourth. Note that for the sake of readability, Fig. 22 only shows the per traffic type results when there are eight, 16 and 24 stations. However, the same results can be derived from the ones presented in Fig. 23.

In line with the above findings, Fig. 23 depicts the evolution over time of the RTS collisions (when RTS/CTS is used) when considering two traffic types. As in the case where a single traffic type was present (Fig. 19), it can be observed that background stations produce a higher number of RTS collisions, due to the long waiting times and the channel usually being occupied by the stations transmitting voice traffic.

## Conclusions and Future Work

In this article, we have presented a configurable and modular CPN model for the IEEE 802.11e protocol. Our proposed model enables the evaluation of the priority and RTS/CTS mechanisms where stations may move from one sector to another. The CPN model enables a fast and seamless configuration of coverage areas, traffic types settings and mobility patterns, as required. As a result, and given the lack of tools providing such evaluation processes and insightful information, this modular CPN model sets the basis for introducing further features (or disable others) to be modeled in wireless networks and to facilitate the analysis and research in the topic.

Throughout our experiments, we have evaluated various scenarios comprising two coverage ranges. From our analysis, we have been able to provide a useful insight into the performance of the stations located in each sector and for each type of traffic. In particular, we have been able to assess the impact of the station mobility and the effectiveness of the RTS/CTS mechanism for each type of traffic. Our results have shown that the RTS/CTS is unable to mitigate the hidden network problem in the case of VO services. This result is highly relevant since it justifies the need for novel access mechanisms for segregating the stations into groups composed of stations capable of sensing each other’s activities. As future work, we plan to extend the analysis to cover the concept of *network slicing* and multi-access edge computing for wireless networks, where the network resources are logically isolated from each other for service-level agreements (SLAs), and a certain level of performance and delay must be ensured for certain network applications.

## Appendix A

Listings 1, 2 and 3 contain the main function declarations used in the CPN model.

      Listing 1: Function declarations (I). 
________________________________________________________________________________________________________ 
fun  dexp( r)=round ( exponential ( r ) ) ; 
fun intTime ()= IntInf . toInt ( time ( ) ) ; 
fun mst ( ap , i )= i f ( ap =0 andalso $i =0 $ ) then 0 else 1; 
fun decr  bo ( n , b )=1 ‘( n , b -1) @ + SlotTime ; 
(* fun change grp ( ng , tg )= 
i f ( uniform (0.0 ,1.0) < pmov ) then 
i f ( uniform (0.0 ,1.0) <0.5) then 1+( ng mod tg ) 
else dec1 ( ng , tg ) 
else ng ; *) 
fun get  type ( n )= i f ( n <= nt1 ) then 1 else 2; 
fun get  n1p ( i )= case i of 
1 => #1 n1p |2 => #2 n1p |3 => #3 n1p |4 => #4 n1p ; 
fun get  n2p ( i )= case i of 
1 => #1 n2p |2 => #2 n2p |3 => #3 n2p |4 => #4 n2p ; 
fun get  prio  aux1 ( n , i )= 
i f ( n <= get  n1p ( i )) then i else get  prio  aux1 ( n - get  n1p ( i ) , i +1); 
fun get  prio  aux2 ( n , i )= 
i f ( n <= get  n2p ( i )) then i else get  prio  aux2 ( n - get  n2p ( i ) , i +1); 
fun get  prio ( n )= 
i f ( n <= nt1 ) then get  prio  aux1 ( n ,1) else get  prio  aux2 ( n - nt1 , 1 ) ; 
fun get  r2 ( n )= case get  prio ( n ) of 
1 => #1 r2 | 2 => #2 r2 | 3 => #3 r2 | 4 => #4 r2 ; 
fun get  Payload ( n )= case get  prio ( n ) of 
1=> #1 Payload | 2 => #2 Payload | 3 => #3 Payload | 4=> #4 Payload ; 
fun get DataRate ( n )= case get  prio ( n ) of 
1 => #1 DataRate | 2 => #2 DataRate | 
3 => #3 DataRate | 4 => #4 DataRate ; 
fun TSEND CTS ( n )=32+ Real . round (( real 384)/( real ( get DataRate ( n ) ) ) ) ; 
(* Time to send a CTS message . n i s the station number *) 
fun TSEND ACK ( n )=32+ Real . round (( real 384)/( real ( get DataRate ( n ) ) ) ) ; ; 
(* Time to transmit ACK . *) 
fun TTIMEOUT ( n )= TSEND ACK ( n ) ; (* Time added to compute time - out *) 
fun paydarate ( n )= Real . round (( (34.0+ ( real ( get  Payload ( n ))))*8.0)/ 
( real ( get DataRate ( n ) ) ) ) ; 
fun get AIFS ( n )= case n of 
1 => #1 AIFS | 2 => #2 AIFS | 3 => #3 AIFS | 4 => #4 AIFS ; 
fun update med ( tr , ap , c )= 
i f ( ap =1 orelse tr >0) then ( tr +1, ap ,1) else ( tr +1, ap , c ) ; 
fun update  bo  time ( n , b , m , a i f s )= 
i f ( $m =1 $ ) then 1 ‘( n , b ) @ +0 else 1 ‘( n , b ) @ + a i f s ; 
fun update  col  end  ack ( s )= i f ( s >=1) then ( s ,0 ,1) else (0 ,0 ,0); 
fun update  col  cts ( s )= i f ( $s >0 $ ) then 1 else 0; 
fun update  col  ack ( tr , c )=  
i f ( $c =1 $ ) then 1 else i f ( tr =0) then 0 else 1; 
fun update  ap  cts ( tr , c )= i f ( tr >0 orelse $c =1 $ ) then 1 else 0; 
__________________________________________________________________________________________________________________________________

                                  Listing 2: Function declarations (II). 
________________________________________________________________________________________________________ 
fun need  backoff ( nb , s , c , sg )= 
i f ( $c >0 $ orelse nb =1 orelse $s >0 $ orelse sg >0) then 1 else 0; 
fun update avg ( i , avg , t , arrival )= 
(( avg *( real i )+ real ( t - arrival )))/( real ( i +1)); 
fun update ap ( tr , ap )= 
i f ( ap =0 andalso tr =1) then (0 ,0 ,0) else 
i f ( ap =0 andalso tr >1) then ( tr -1 ,0 ,1) else ( tr -1, ap , 1 ) ; 
fun new imm ( n )= 
i f ( n <= nt1 ) then 1 ‘ n else n i l ; 
fun gen new ( n )= i f ( n > nt1 ) then dexp ( get  r2 ( n )) else dexp ( r1 ) ; 
fun gen new time ( n )= 
i f ( n > nt1 ) then 1 ‘ n@ + dexp ( get  r2 ( n )) else n i l ; 
fun get CWMIN ( i )= case i of 
1 => #1 CWMIN |2 => #2 CWMIN | 
3 => #3 CWMIN |4 => #4 CWMIN ; 
fun get CWMAX ( i )= case i of 
1 => #1 CWMAX |2 => #2 CWMAX | 
3 => #3 CWMAX |4 => #4 CWMAX ; 
fun tsend ( n )=32+ paydarate ( n ) ; 
fun tsendRTS ( n )=32+ Real . round (( real 384)/( real ( get DataRate ( n ) ) ) ) ; 
fun timeout ( n )= tsend ( n )+2* SIFS + TSEND ACK ( n )+ TTIMEOUT ( n ) ; 
(* microseconds after ACK was expected *) 
fun toutcts ( n )= tsend ( n )+2* SIFS + TSEND ACK ( n )+1; 
fun M NULL ( n )= i f ( $n >0 $ ) then 1 ‘( n ,0)++ M NULL ( n -1) else n i l ; 
fun p2 ( bc )= i f ( bc =0) then 1 else 
i f ( bc >10) then 1024 else 2* p2 ( bc -1); 
fun get cw ( bc , cwmin )=( cwmin )* p2 ( bc ) ; 
fun M ICONF ( n )= 
i f ( $n >0 $ ) then 1 ‘( n ,0,0)++ M ICONF ( n -1) else n i l ; 
fun M MSG ( n )= 
i f ( $n >0 $ ) then 1 ‘ n@ + gen new ( n )+++ M MSG ( n -1) else n i l ; 
fun M TT ( n )= 
i f ( $n >0 $ ) then 1 ‘( n ,0 ,0.0)++ M TT ( n -1) else n i l ; 
fun new  arrival ( n )= 
i f ( n <= nt1 ) then 1 ‘( n ,0 ,1) else 1 ‘( n , 0 , 0 ) ; 
fun new  i  delayed ( n )= 
i f ( n <= nt1 ) then 1 ‘ n@ + dexp ( r1 ) else n i l ; 
fun TOUT CTS ( n )= tsendRTS ( n )+ TSEND CTS ( n )+ TTIMEOUT ( n ) ; 
(* Time that other stations wait after receiving CTS *) 
fun M TCTS ( n )= i f ( $n =1 $ ) then 1 ‘1 @0 else 1 ‘ n@0 +++ M TCTS ( n -1); 
fun nt  tokens ( n )= i f ( $n =1 $ ) then 1 ‘1 else 1 ‘ n ++ nt  tokens ( n -1); 
(* Update time of a l l stations but n1 , in order to retransmit 
after a CTS was received *)  
fun nt  new  tokens ( n , n1 )= 
i f ( $n =1 $ ) then i f ( n1 =1) then 1 ‘1 @ +0 else 
1 ‘ n@ + toutcts ( n1 ) else 
i f ( n = n1 ) then 1 ‘ n@ +0+++ nt  new  tokens ( n -1, n1 ) 
else 1 ‘ n@ + toutcts ( n1 ) +++ nt  new  tokens ( n -1, n1 ) ; 
__________________________________________________________________________________________________________________________________

                                 Listing 3: Function declarations (III). 
________________________________________________________________________________________________________ 
fun   get _group _init (n)= 
i f ( nt mod 2 = 0) then 
i f ( n <= ( nt div 2)) then 1 else 2 
else 
i f ( n <= (( nt +1) div 2)) then 1 else 2; 
fun get  group  init  rnd ( n , m1 , m2 )= 
i f ( m1 =0) then 2 
else i f ( m2 =0) then 1 
else discrete (1 ,2); 
fun num g1 ( n )= 
i f ( n mod 2 = 0) then n div 2 
else ( n +1) div 2; (* I n i t i a l number of st in group 1 *) 
fun num g2 ( n )= n - num g1 ( n ) ; (* I n i t i a l number in group 2 *) 
fun M STCONF ( n , m1 , m2 )= 
i f ( $n >0 $ ) then 
i f ( get  group  init  rnd ( n , m1 , m2 )=1) then 
1 ‘( n , get AIFS ( get  prio ( n )) , get CWMIN ( get  prio ( n )) , 
get CWMAX ( get  prio ( n )),1)++ M STCONF ( n -1, m1 -1, m2 ) 
else 1 ‘( n , get AIFS ( get  prio ( n )) , get CWMIN ( get  prio ( n )) , 
get CWMAX ( get  prio ( n )),2)++ M STCONF ( n -1, m1 , m2 -1) 
else n i l ; 
fun MOVINIT ( n )= 
( num g2 ( n ) , 1); (* Stations to move and cycle 1 *) 
fun movs ( n )= 
case n of 
1 => #1 changes  stgrp |2 => #2 changes  stgrp | 
3 => #3 changes  stgrp | 4 => #4 changes  stgrp ; 
fun movst1 ( n , s )= 
case s of 
1 => #1 ( movs ( n )) | 2 => #2 ( movs ( n )) | 
3 => #3 ( movs ( n )) | 4 => #4 ( movs ( n ) ) ; 
fun movst ( n , s ) = 
i f ( $n =0 $ ) then 0 else 
i f ( $s >4 $ ) then 0 else 
i f ( n <= 4) then movst1 ( n , s ) else 1+ movst ( n -4, s ) ; 
(* number of stations that go to group 1 , now at cycle s *) 
fun ischange ( n , s )= 
( movst ( num g2 ( n ) , s ) <> 0); (* stations move ? *) 
__________________________________________________________________________________________________________________________________

## Additional Information and Declarations

### Data Availability

The following information was supplied regarding data availability: The data is available at Zenodo: María Emilia Cambronero. (2022). Modelling, Simulation and Performance Evaluation of the IEEE 802.11e Protocol with Station Mobility. https://doi.org/10.5281/zenodo.7665577.
